# The Metabolomics Society—Current State of the Membership and Future Directions

**DOI:** 10.3390/metabo9050089

**Published:** 2019-05-03

**Authors:** Krista A. Zanetti, Robert D. Hall, Julian L. Griffin, Sastia Putri, Reza M. Salek, Mark P. Styczynski, Fidele Tugizimana, Justin J.J. van der Hooft

**Affiliations:** 1 Epidemiology and Genomics Research Program, Division of Cancer Control and Population Sciences, National Cancer Institute, National Institutes of Health, Rockville, MD 20892, USA; 2 Laboratory of Plant Physiology, Wageningen University and Research, BU Bioscience, 6708 PB Wageningen, The Netherlands; robert.hall@wur.nl; 3 Department of Biochemistry and Cambridge Systems Biology Centre, University of Cambridge, Cambridge CB21GA, UK; Jlg40@cam.ac.uk; 4 Department of Biotechnology, Graduate School of Engineering, Osaka University, Osaka 565-0871, Japan; sastia_putri@bio.eng.osaka-u.ac.jp; 5 International Agency for Research on Cancer, 69372 Lyon CEDEX 08, France; SalekR@iarc.fr; 6 School of Chemical and Biomolecular Engineering, Georgia Institute of Technology, Atlanta, GA 30332, USA; mark.styczynski@chbe.gatech.edu; 7 Centre for Plant Metabolomics Research, Department of Biochemistry, University of Johannesburg, Johannesburg 2006, South Africa; ftugizimana@uj.ac.za; 8 International Research and Development, Omnia Group, Ltd., Bryanston Johannesburg 2021, South Africa; 9 Bioinformatics Group, Plant Sciences Group, Wageningen University, 6708 PB Wageningen, The Netherlands; justin.vanderhooft@wur.nl

**Keywords:** Metabolomics Society, membership, metabolomics, survey

## Abstract

*Background:* In 2017, the Metabolomics Society conducted a survey among its members to assess the degree of its current success, define opportunities for improving its service to the community and make plans to establish future goals and direction of the Society. *Methods:* A 32-question online survey was sent via e-mail to all Metabolomics Society members as of 19 June 2017 (n = 644). In addition to the direct e-mails, the link to access the survey was made available through social media. The survey was open until 10 August 2017. Question-specific data were reported using the summary data generated by SurveyMonkey and additional stratified analyses performed using Stata 15. *Results:* The number of respondents was 394 (61%) with 348 (88%) completing the multiple-choice questions in survey. Metabolomics Society annual meetings, networking and the opportunity to join the global metabolomics community were among the most important benefits expressed by the Metabolomics Society members. *Conclusions:* The survey collected the first data focusing on membership issues from Society members. The Society should focus on collecting and monitoring of demographic data during the membership registration process; continuing to support the early-career members of the Society; and developing initiatives that focus on member networking to retain and increase Society membership.

## 1. Introduction

The Metabolomics Society is an independent, non-profit organization comprising dedicated members of the metabolomics community who are committed to the establishment and adoption of metabolomics approaches in the life sciences. The Society’s founding vision was to become the premier organization devoted to supporting the development of metabolomics-based research, define standard practices and encourage cross-disciplinary collaborations to establish sustainable robust approaches. The society was constituted in 2004 and membership has grown to over 1000 scientists from more than 40 countries in 15 years’ time. Membership registration is directly linked to the annual meeting registration, so yearly membership varies depending on where the annual meeting is being held.

The Society’s initiation represented a logical progression of the accelerating interest in metabolomics applications across all biological fields, including plant, animal, microbial, and human research, since the establishment of the technology in the first years of this century. The Society brings together scientists from all relevant disciplines representing metabolomics, metabonomics, metabolite target analysis, metabolic profiling, metabolic fingerprinting, metabolic flux analysis, analytical chemistry, biochemical modelling, chemometrics, epidemiology, statistics, and related informatics fields. It has been highly active in defining research best practices, recommending publishing standards [1,2,3,4] and establishing a dedicated journal for metabolomics-based technological and biological research. The Society has also established several Task Groups to address specific needs within the field (http://metabolomicssociety.org/). The Society’s mission is to promote (1) the growth and development of the field of metabolomics internationally, (2) opportunities for collaboration and association between academia, government and industry in the field of metabolomics, (3) opportunities for conferences and workshops, and (4) the publication of meritorious research in the field.

Developments within the omics fields are rapid and hence the Society must co-evolve to meet current and future needs. Furthermore, the Society has a great desire to reach out to an even broader community. For example, several data standards activities and computational biology initiatives are jointly held with the Proteomics Standards Initiative community, as they share underlying mass spectrometry technology. In addition, metabolite identification efforts can benefit from close working relationships with the natural product chemistry and cheminformatics societies. Thus, there is a need to reach out to new interested parties across analytical and biological sciences.

In 2017, the Society executed a broad survey of its members to assess the degree of its current success in achieving its mission, define opportunities for improving its service to the community, and make plans to establish the future goals and direction of the Society. Through this short summary, we present the main findings of this evaluation, provide access to the results and make key conclusions defining how the Society aims to move forward in its quest to continue to support community needs and desire to promote metabolomics.

## 2. Results

### 2.1. Survey Response Rates

The total number of respondents to the survey was 394, which is a 61% response rate based on the Metabolomics Society having 644 members as of 19 June 2017. Of the 61% who responded, 348 completed the survey (88%). The response rates were higher for the questions at the beginning of the survey (100%) and declined for later questions. The final multiple-choice question had a completion rate of 90%. The open-ended questions at the end of the survey had a much lower response rate (28% and 31%). SurveyMonkey (https://www.surveymonkey.com/) reported that the estimated time to complete the survey was an average of 12 min.

### 2.2. Membership Demographics

**Figure 1:**
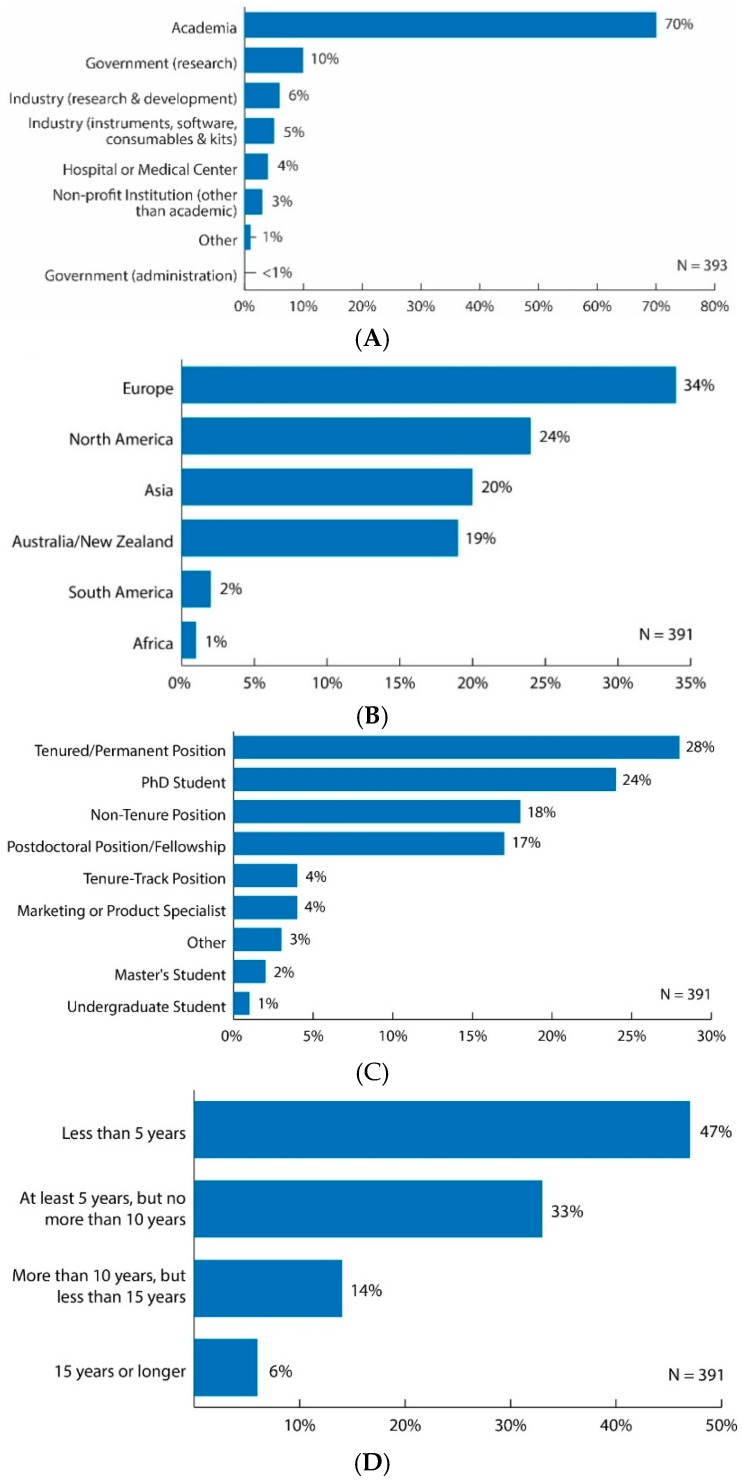
(**A**) Bar graph of survey results for question: “In what type of institution do you work?” Original data used to create the figure can be found in the supplementary information under question number 1; (**B**) Bar graph of survey results for question: “In what geographical region is your main research operation?” Original data used to create the figure can be found in the supplementary information under question number 2; (**C**) Bar graph of survey results for question: “Which best describes your current professional status?” Original data used to create the figure can be found in the supplementary information under question number 4; (**D**) Bar graph of survey results for question: “How long have you been working in the field of metabolomics?” Original data used to create the figure can be found in the supplementary information under question number 5.

Approximately 47% of respondents were female and 51% were male, while a small percentage of respondents preferred not to answer this question (see supplementary information). Most respondents hold positions at an academic institution (70%) (Figure 1A). Both the government sector (research and administration) and industry each represented 11% of respondents, with industry being almost evenly split between instruments, software, consumables and kits (5%) and research and development (6%). Four percent of respondents held positions at hospitals or medical centers and 3% at non-profit institutions.

The primary geographical regions in which respondents conducted their main research operations were Europe (34%), North America (24%), Asia (20%) and Australia/New Zealand (19%) (Figure 1B). Significantly fewer respondents had their main research operation in South America (2%) and Africa (1%) (Figure 1B). More specific data on the country of respondents’ main research operation were collected under question 3 of the survey: Please list the country of your main research operation.

Most respondents described their professional status as either tenured/permanent positions (28%), PhD students (24%), non-tenure positions (18%) or postdoctoral fellows (17%) (Figure 1C). The remaining respondents’ professional status was reported as tenure-track positions (4%), marketing or product specialists (4%), other (3%), master’s students (2%) and undergraduate students (1%) (Figure 1C). Therefore, 54% of respondents were in non-trainee/senior positions (tenured/permanent positions, non-tenure positions, tenure-track positions and marketing or product specialists) and 44% of respondents were in trainee positions (postdoctoral fellows, PhD students, master’s students, and undergraduate students).

Eighty percent of respondents had been in the field of metabolomics for less than 10 years (Figure 1D). More specifically, 47% of the respondents had been working in the field less than 5 years; 33% for at least 5 years, but no more than 10 years; 14% for more than 10 years, but less than 15 years; and 6% for 15 years or longer.

### 2.3. Society Annual Meeting

Seventy-two percent of respondents had attended at least one Metabolomics Society Annual Meeting. However, only 38% remained a Society member in the years that they do not attend the annual meeting; whereas 16% sometimes remained a member. Of those respondents that had attended a meeting, most had attended the 2017 Brisbane, AU conference (76%), followed by the 2016 Dublin, IE meeting (43%) and 2015 San Francisco, USA meeting (34%), down to 4% in Tsuruoka, Japan in 2005 (see supplementary information). The primary reasons indicated for not attending the Society’s annual meeting were that no travel funds were available (52%), the cost is too high (32%), and the locations are not convenient (25%) (see supplementary information).

Thirty-nine percent (n = 152) of all survey respondents answered the question “When you attended annual Metabolomics Society meeting(s), did you also attend any of the Workshops, which generally take place the day prior to the official start of the meeting?” Of those respondents, approximately 41% attended the workshops regularly and 26% attended sporadically; whereas 13% of respondents had attended one workshop and 19% had not attended any. Of those who attended a workshop(s) (n = 121), 85% believed it was clear that the purpose of the workshops was training (see supplementary information).

### 2.4. Society Membership Opportunities

**Figure 2:**
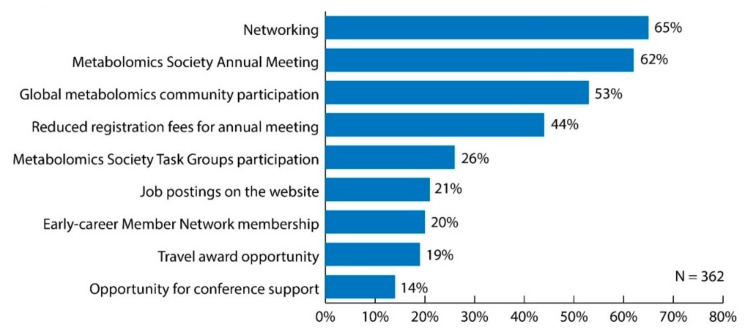
Bar graph of survey results for question: “What do you consider to be the most important benefits of being a Metabolomics Society member? Choose all that apply.” Original data used to create the figure can be found in the supplementary information under question number 16.

When asked what the most important benefits of being a Metabolomics Society member are, over 50% of respondents selected networking (65%), the Metabolomics Society annual meeting (62%), and the opportunity to join the global metabolomics community (53%) (Figure 2). When examining data specific only to respondents working in industry positions (n = 43), these three benefits were also identified as the most important. When stratifying by professional status, those initiatives/activities that differed more than 10% between respondents holding senior positions versus trainee positions included job postings on the website (12% senior versus 35% trainee), opportunity to apply for a travel award (6% senior versus 36% trainee), opportunity to apply for conference support (9% senior versus 20% trainee), and membership in the Early-career Member Network (EMN) (10% senior versus 34% trainee).

**Figure 3:**
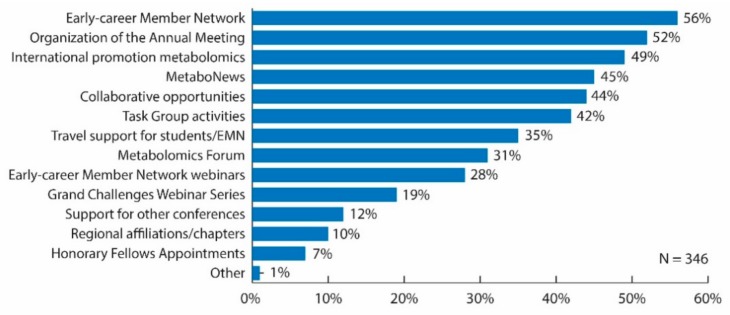
Bar graph of survey results for question: “What do you believe are the most important initiatives/activities that are currently supported by Metabolomics Society? Choose all that apply.” Original data used to create the figure can be found in the supplementary information under question number 18.

Over 50% of respondents deemed the most important initiatives/activities currently supported by the Metabolomics Society to be the Early-career Member Network (56%) and organization of the annual meeting (52%). There were also several initiatives/activities that over 40% of respondents considered to be the most important initiatives/activities currently supported by the Metabolomics Society, including the promotion of the field of metabolomics internationally (49%), MetaboNews (45%), collaborative opportunities (44%), and Task Group activities (42%) (Figure 3). When examining the data for only those respondents working in industry positions, this sector of the membership felt that most important initiatives/activities currently supported by the Metabolomics Society were the promotion of the field of metabolomics internationally (55%), organization of the Annual Meeting (50%), and the Early-career Member Network (45%). When these results were stratified by professional status, the initiatives/activities that differed by more than 10% between senior and trainee respondents included: promotion of the field of metabolomics internationally (58% senior versus 37% trainee); organization of the Annual Meeting (57% senior versus 44% trainee); MetaboNews (53% senior versus 36% trainee); travel support for students/EMN (28% senior versus 47% trainee); and the EMN webinar series (23% senior versus 36% trainee).

**Figure 4:**
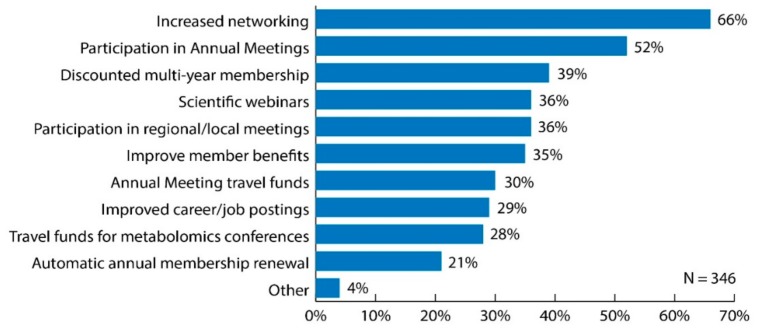
Bar graph of survey results for question: “What are the best strategies for the Metabolomics Society to maintain members? Choose all that apply.” Original data used to create the figure can be found in the supplementary information under question number 19.

Sixty-six percent of respondents considered the best strategy for the Metabolomics Society to maintain members to be increased networking among members (Figure 4). Increased opportunities to participate in annual meetings was selected by over 50% of respondents (52%); whereas less than 40% of respondents selected the other options (Figure 4). Examining these data to include only responses from those working in industry positions, increased networking among members was identified as the best strategy to maintain members (68%); whereas less than 40% of those members identified increased opportunities to participate in the annual meeting as one of the best strategies. Those initiatives/activities that differed by professional status more than 10% included opportunity to apply for travel funds to annual meeting (21% senior versus 42% trainee); opportunity to apply for travel funds to any metabolomics conference/meeting (21% senior versus 37% trainee); and improved career/job postings (20% senior versus 41% trainee).

**Figure 5:**
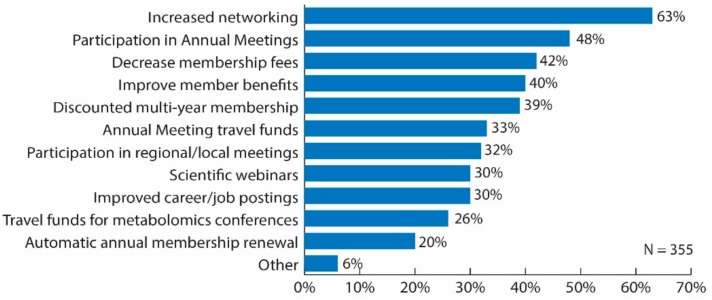
Bar graph of survey results for question: “What are the best strategies for the Metabolomics Society to increase membership? Choose all that apply.” Original data used to create the figure can be found in the supplementary information under question number 20.

Sixty-three percent of respondents considered the best strategy for the Metabolomics Society to increase members to be through increased networking among its members (63%) (Figure 5). Additionally, increased opportunities to participate in annual meetings was selected by 48% of respondents, decreased membership fees by 42% and to improve member benefits by 40%; whereas less than 40% of respondents selected the other options (Figure 5). When examining the data specific to respondents working in industry positions, networking among members was the considered the best strategy to increase membership (69%); whereas less than 40% of those respondents selected any of the other options. When stratifying the data by professional status, those initiatives/activities that differed by more than 10% between senior and trainee included a discounted multi-year membership option (44% senior versus 33% trainee); opportunity to apply for travel funds to Annual Meeting (28% senior versus 40% trainee); and improved career/job postings (20% senior versus 43% trainee).

## 3. Discussion

The purpose of the 32-question Metabolomics Society membership survey was to gather data to help guide the future of the Society. The survey was completed by a significant proportion of Society members, which suggests that the results can be reliably extrapolated to the larger Society membership. The survey had an equivalent number of responses from males and females, as well as a substantial number of respondents who are in trainee positions. However, one caveat is that the survey was administered in conjunction with the Brisbane, AU annual meeting. It is possible that the demographics of the respondents would have differed had the survey been administered in conjunction with an annual meeting that was held in Europe or North America.

The Society collects very minimal information at registration that includes no demographic information other than if the registrant qualifies as an early-career member, which is defined as being within five years of the attainment of a graduate degree. Therefore, the Society should consider collecting additional demographic data that are asked of all members upon registration, while still allowing any member to opt out of providing personal information if desired. Having sufficient member demographic data would position the Society better to determine what initiatives would best serve the membership and track trends over time. Furthermore, it would allow the Society to establish targeted recruitment efforts and determine if those efforts were meeting the established goals. Based on the survey, the Society should be targeting increased membership recruitment in Africa and South America; however, without collecting these data from member registrations, it won’t be possible to evaluate whether recruitment effort initiatives are successful. Given the size of these geographical areas, it might also be valuable to further stratify for regions within these continents to better target recruitment efforts.

Seventy percent of respondents held positions in an academic institution; whereas 11% of respondents held industry positions. Although many of the responses by those working in industry aligned with the responses of the broader membership, some differences were noted. These differences suggest that Society members working in industry positions may have different interests or needs in specific areas. The Society recently revitalized the Industry Engagement Task Group, which includes members who hold positions in industry along with some members in non-industry positions, to address industry-specific interests and needs.

The survey data also showed that a significant portion of the Society membership was in training positions. Additionally, 80% of respondents had been in the field less than 10 years. Forty-seven percent of those respondents had been working in the field less than 5 years, which primarily reflects early-career members (data not shown). This suggests the still nascent nature of metabolomics and the continued adoption of the technology by additional research groups. To support this sector of the membership, the Society established the Early-career Member Network (EMN) Committee in 2013 to provide a forum for metabolomics researchers at the start of their professional career and serve the early-career members of the Metabolomics Society (http://metabolomicssociety.org/board/society-committees/early-career-members-network-emn-committee). The EMN Committee has several ongoing projects and initiatives including a webinar series, bursary program for early-career members to travel to conferences, and organization of workshops and a reception at the Annual Meeting. Additionally, members of the EMN Committee participate on several Society Committees and Task Groups, and the Chair of the EMN Committee holds a position on the Society Board of Directors. Survey respondents considered one of the most important initiatives/activities currently supported by the Metabolomics Society to be the EMN.

Seventy-two percent of respondents had attended a Metabolomics Society Annual Meeting; however, only 38% remained a Society member in the years that they do not attend the annual meeting. This suggests that the Society should develop additional incentives to retain members even when they do not attend the annual meeting. Respondents felt the best strategies to maintain the current membership to be increased networking among members and increased opportunities to participate in annual meetings, which provides members an opportunity to network. Furthermore, respondents considered the best strategy to increase membership to be increased networking among members. This was consistent regardless of professional status (data not shown); therefore, increased opportunities to network across the broad membership should be explored to both maintain and increase membership. Several activities have recently been held at the annual meeting to increase networking opportunities. The EMN holds a reception for early-career members to network with their peers and the Board of Directors. Additionally, regional affiliates have held receptions to encourage those in geographical proximity to each other to network and join their local affiliates. Based on the survey, these types of activities are in line with what the membership regards as the most important strategy to maintain and increase Society membership. However, additional networking opportunities should be explored and established for the full membership and in conjunction with the Society’s regional affiliates (http://metabolomicssociety.org/international-affiliations/current-affiliates).

Although trainees agreed with the broader membership that networking and the annual meeting were the most important benefits of being a Society member, they held career development opportunities as being more important than the senior, more established members of the Society. Specifically, those initiatives/activities that were more important to trainees were job postings on the website, opportunities to apply for travel awards, opportunities to apply for conference support, and membership in the EMN. The Society provides travel awards to the annual meeting for early-career members. Additionally, in 2018, the EMN established a bursary program to support increased participation of early-career researchers at both the Society’s annual meeting and other metabolomics-focused conferences. The Society should review the current career-development focused initiatives and consider expanding them to include additional support.

The broad Society membership agrees that the EMN and organization of the annual meeting are key initiatives/activities currently supported by the Metabolomics Society; whereas the senior members also value promotion of the field internationally and publication of MetaboNews. MetaboNews is a newsletter that is published in partnership between the Metabolomics Innovation Centre (Canada) and the Metabolomics Society to keep metabolomics researchers and other professionals informed about a number of topics in the field (http://www.metabonews.ca/archive.html), which is an opportunity to promote the field internationally. The Society should liaise with the editor of MetaboNews to identify ways to further promote the field through this online publication. They additionally should work to identify other ways for international promotion, possibly by reaching out to South American and African members who have the least Society representation, as well as work with our established international affiliates.

Overall, based on the survey results, areas for the Society to focus on to support community needs are:

- collecting and monitoring of demographic data during the membership registration process;
- continuing to support the early-career members of the Society through initiatives such as the EMN;
- developing initiatives that focus on member networking to retain and increase Society membership, including increased interaction with the Society’s local affiliates to identify specific needs for various geographical regions.

## 4. Materials and Methods

A survey of 32 questions, 5 of which were open-ended, was created in SurveyMonkey (https://www.surveymonkey.com/; see supplementary information). It was sent via e-mail on June 24, 2017 to all Metabolomics Society members paid through 19 June 2017. Follow-up e-mails were sent to non-responders on 11 July 2017 (n = 495), 18 July 2017 (n = 430), and 25 July 2017 (n = 384). Ultimately, 297 members responded to the direct e-mail contact. For those who did not wish to be contacted following the first e-mail message, there was an option to unsubscribe.

In addition to the direct e-mails, the link to access the survey was posted on the Metabolomics Society website (http://metabolomicssociety.org/) from 24 June 2017 to 10 August 2017. It was posted in MetaboNews (http://www.metabonews.ca/archive.html) in the July and August issues, on the Metabolomics Society Facebook page on 25 June 2017 (3 shares); on the Early-career Member Network of the Metabolomics Society Facebook page on 11 July 2017 (0 shares), 18 July 2017 (0 shares), and 26 July 2017 (3 shares); and on the Metabolomics Society Twitter feed on 25 June 2017 (9 retweets), 13 July 2017 (9 retweets), and 8 August 2017 (2 retweets). Ninety-six members responded to the links on the website, Facebook, Twitter, Metabolomics Society Forum and in MetaboNews. The survey closed on 10 August 2017.

Members were incentivized to participate in the survey with a random chance to win a $200 Amazon gift card selected from all members who answered the last question on the survey: If you would like to be entered to win a $200 Amazon gift card, please provide your e-mail address. The number of gift cards was dependent upon the number of respondents. For every 100 respondents, one gift card was given away in the random drawing. Because there were 393 respondents, three Amazon gifts cards were given away using a random number generator, where those with respondents assigned the generated numbers 1, 2, and 3 were awarded the gift certificates.

Question-specific data were reported using the summary data generated by SurveyMonkey and additional stratified analyses performed using Stata 15.

## Supplementary Materials

The following are available online at https://www.mdpi.com/2218-1989/9/5/89/s1, Supplementary Information: The survey summary data generated by SurveyMonkey are reported. These data include only the summary data of the multiple-choice questions. The open-ended questions are not included in the summary data because they are individual response data.
