# Supplementary material for: The Metabolomics Society—Current State of the Membership and Future Directions
Source: Metabolites. 2019 May 3;9(5):89. doi: 10.3390/metabo9050089 (PMC6572628; doi:10.3390/metabo9050089)
Supplement: Supplementary file 1 [file metabolites-09-00089-s001.pdf]

Q1 In what type of institution do you work?

Answered: 393 Skipped: 0

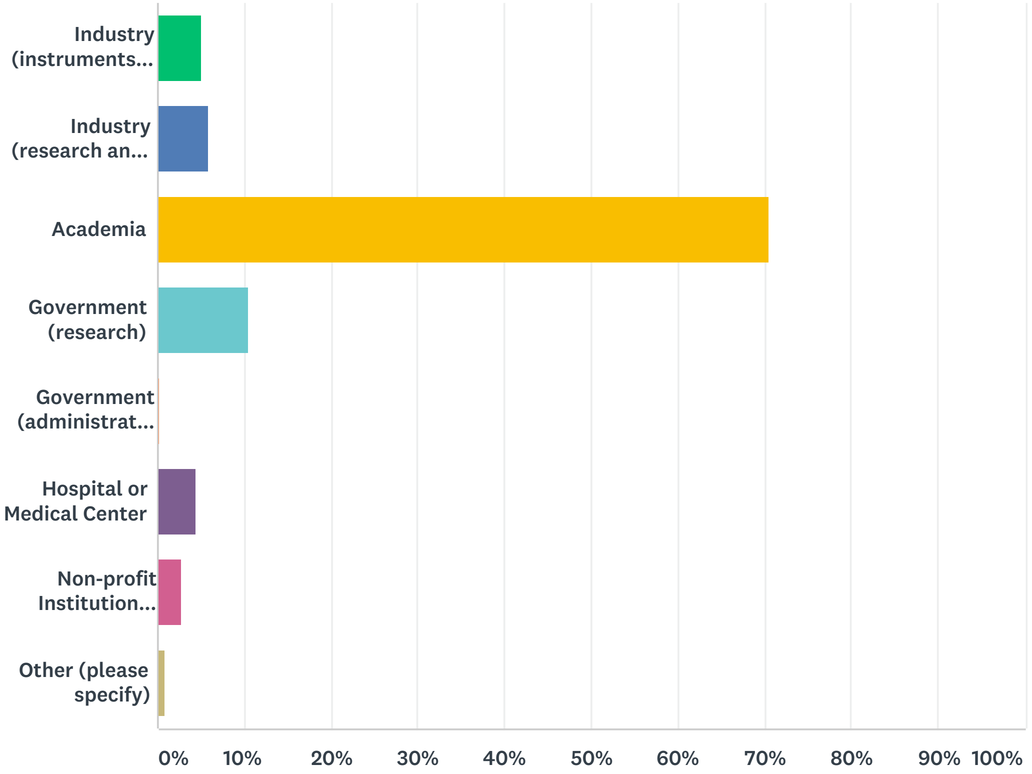

ANSWER CHOICES	RESPONSES	
Industry (instruments, software, consumables and kits)	5.09%	20
Industry (research and development)	5.85%	23
Academia	70.48%	277
Government (research)	10.43%	41
Government (administration)	0.25%	1
Hospital or Medical Center	4.33%	17
Non-profit Institution (other than academic)	2.80%	11
Other (please specify)	0.76%	3
TOTAL		393

Q2 In what geographical region is your main research operation?

Answered: 391 Skipped: 2

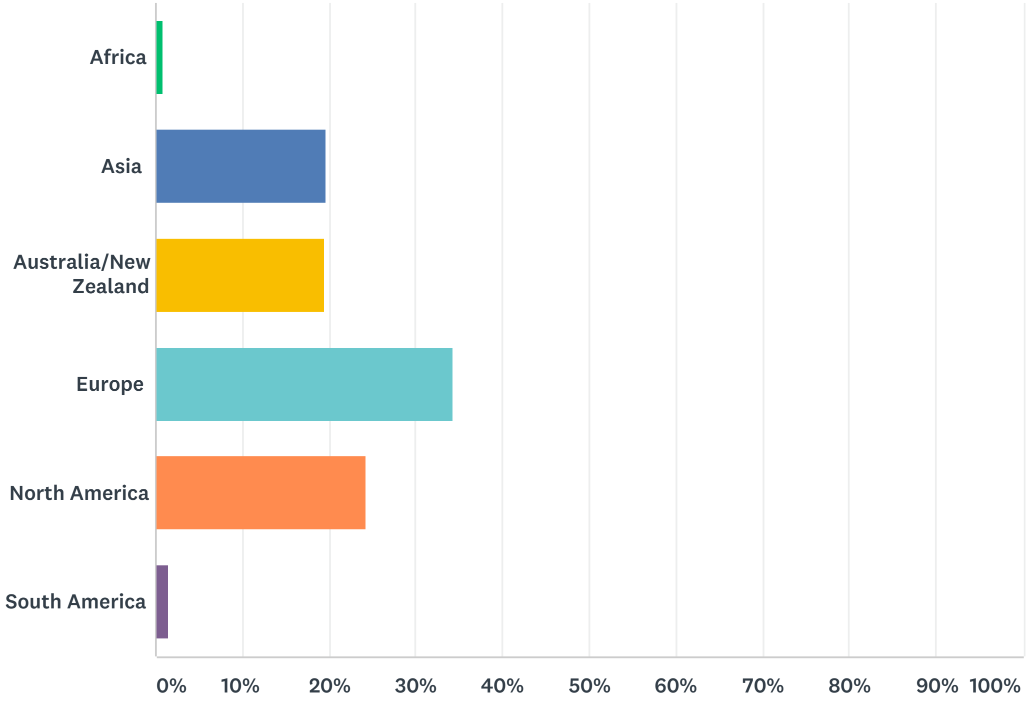

ANSWER CHOICES	RESPONSES	
Africa	0.77%	3
Asia	19.69%	77
Australia/New Zealand	19.44%	76
Europe	34.27%	134
North America	24.30%	95
South America	1.53%	6
TOTAL		391

Q3 Please list the country of your main research operation.

Answered: 355 Skipped: 38

Q4 Which best describes your current professional status?

Answered: 391 Skipped: 2

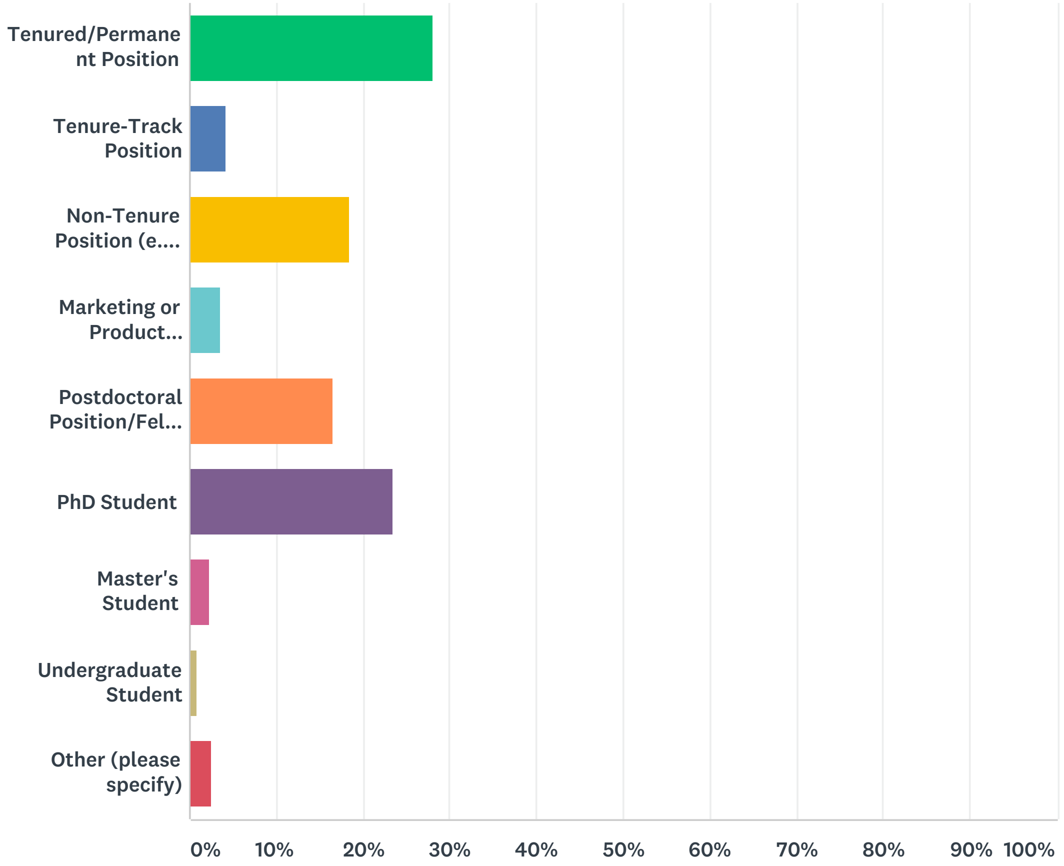

ANSWER CHOICES	RESPONSES	
Tenured/Permanent Position	28.13%	110
Tenure-Track Position	4.09%	16
Non-Tenure Position (e.g. staff scientist, contract appointment)	18.41%	72
Marketing or Product Specialist	3.58%	14
Postdoctoral Position/Fellowship	16.62%	65
PhD Student	23.53%	92
Master's Student	2.30%	9
Undergraduate Student	0.77%	3
Other (please specify)	2.56%	10
TOTAL		391

Q5 How long have you been working in the field of metabolomics?

Answered: 391 Skipped: 2

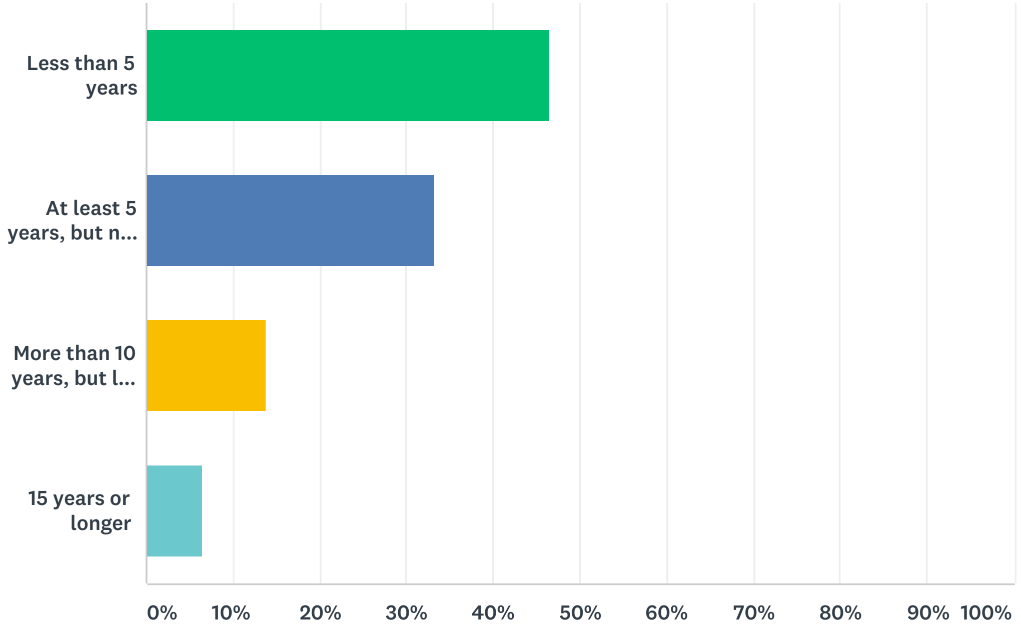

ANSWER CHOICES	RESPONSES	
Less than 5 years	46.55%	182
At least 5 years, but no more than 10 years	33.25%	130
More than 10 years, but less than 15 years	13.81%	54
15 years or longer	6.39%	25
TOTAL		391

Q6 What is your gender?

Answered: 392 Skipped: 1

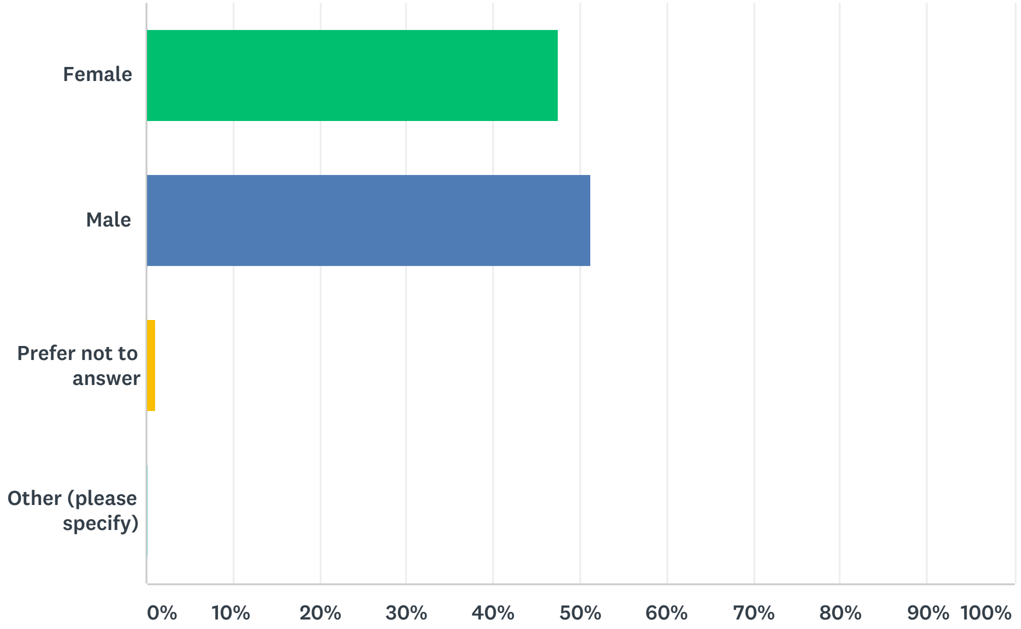

ANSWER CHOICES	RESPONSES	
Female	47.45%	186
Male	51.28%	201
Prefer not to answer	1.02%	4
Other (please specify)	0.26%	1
TOTAL		392

Q7 In what area(s) of science are you currently applying metabolomics? Choose all that apply.

Answered: 391 Skipped: 2

Metabolomics Society Membership Survey

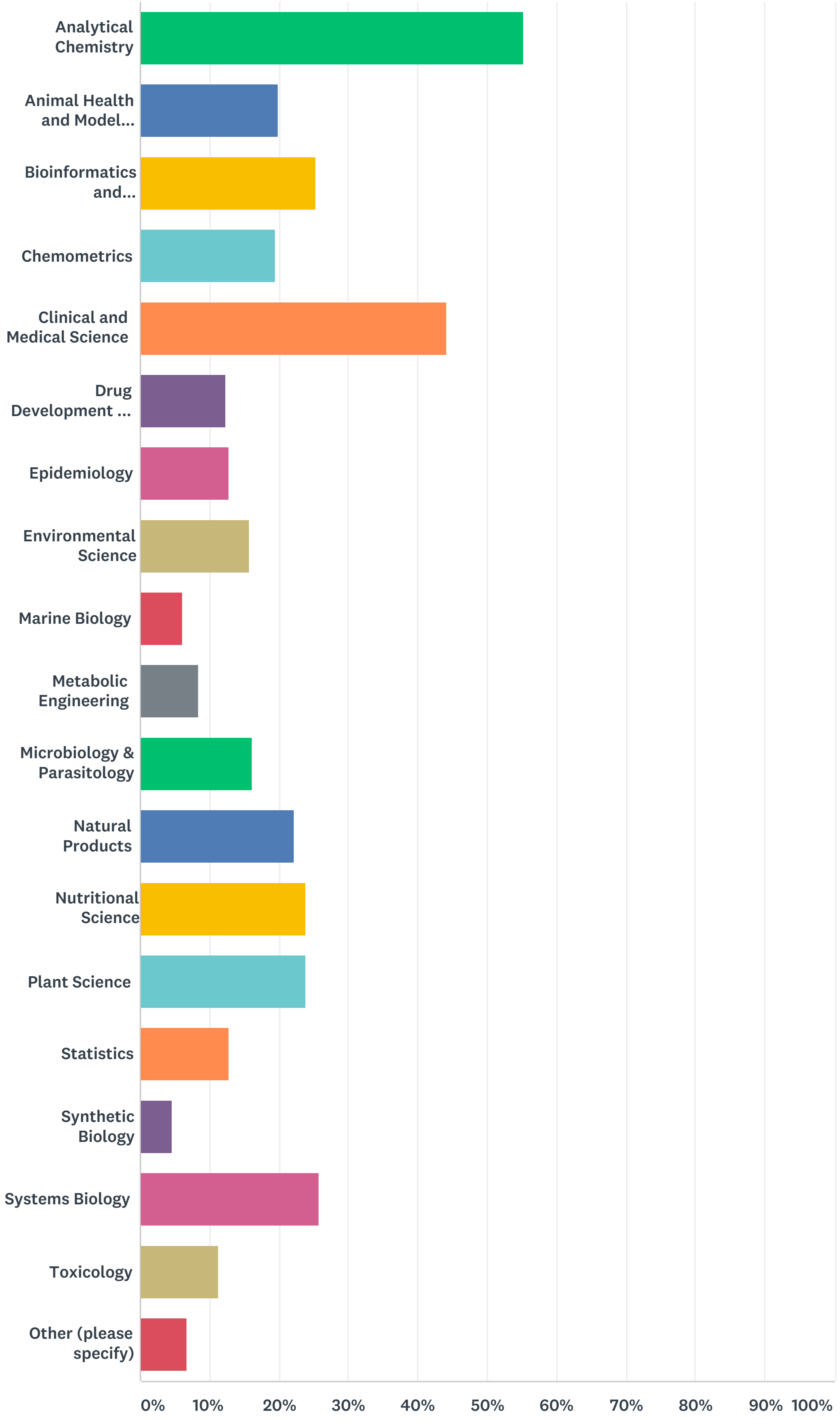

ANSWER CHOICES	RESPONSES

Analytical Chemistry	55.24%	216
Animal Health and Model Systems	19.95%	78
Bioinformatics and Computational Biology	25.32%	99
Chemometrics	19.44%	76
Clinical and Medical Science	44.25%	173
Drug Development and Discovery	12.28%	48
Epidemiology	12.79%	50
Environmental Science	15.60%	61
Marine Biology	6.14%	24
Metabolic Engineering	8.44%	33
Microbiology & Parasitology	16.11%	63
Natural Products	22.25%	87
Nutritional Science	23.79%	93
Plant Science	23.79%	93
Statistics	12.79%	50
Synthetic Biology	4.60%	18
Systems Biology	25.83%	101
Toxicology	11.25%	44
Other (please specify)	6.65%	26
Total Respondents: 391		

Q8 When did you first join the Metabolomics Society?

Answered: 377 Skipped: 16

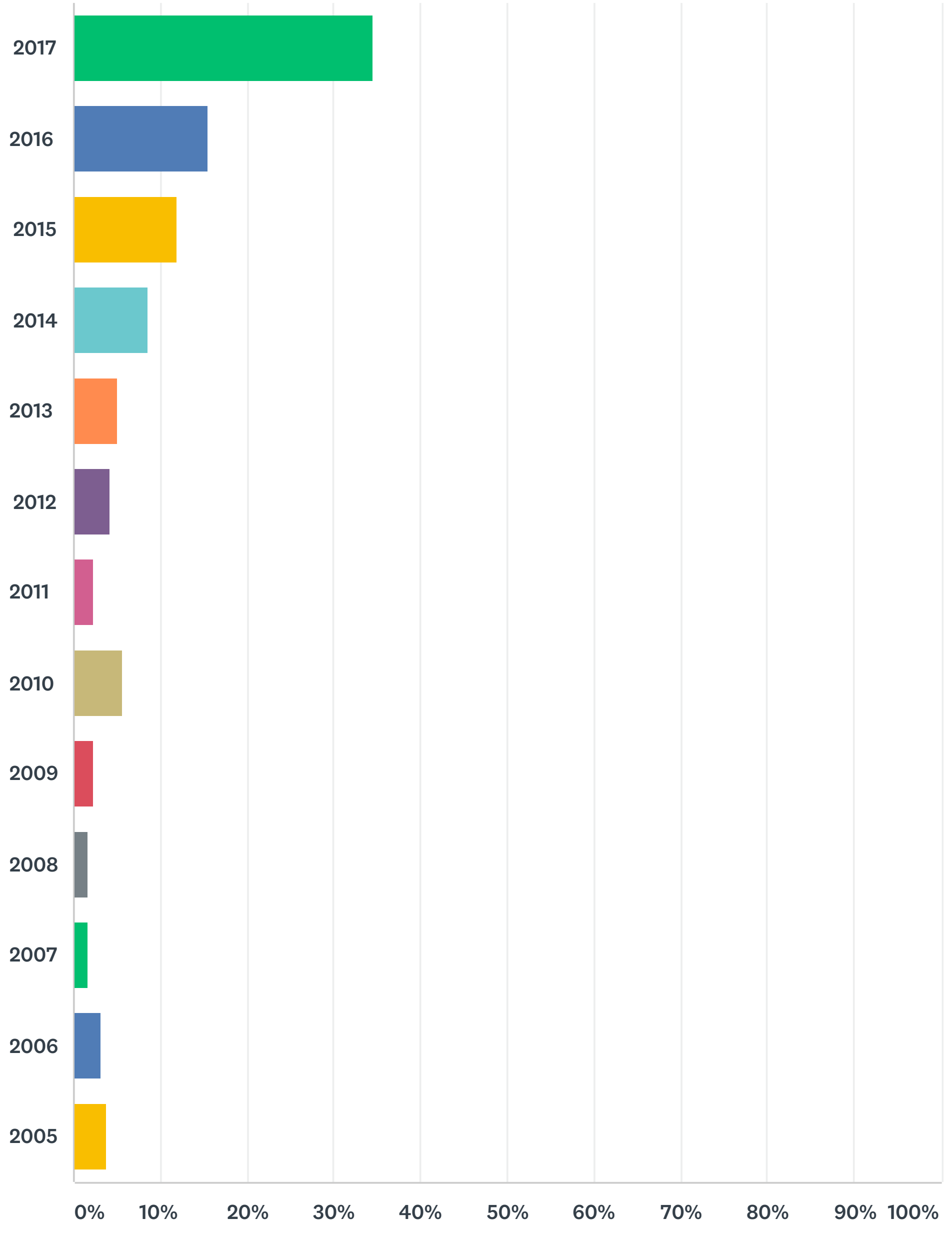

ANSWER CHOICES	RESPONSES	
2017	34.48%	130
2016	15.38%	58
2015	11.94%	45
2014	8.49%	32
2013	5.04%	19
2012	4.24%	16

2011	2.39%	9
2010	5.57%	21
2009	2.39%	9
2008	1.59%	6
2007	1.59%	6
2006	3.18%	12
2005	3.71%	14
TOTAL		377

Q9 Have you been to a Metabolomics Society Annual Conference?

Answered: 391 Skipped: 2

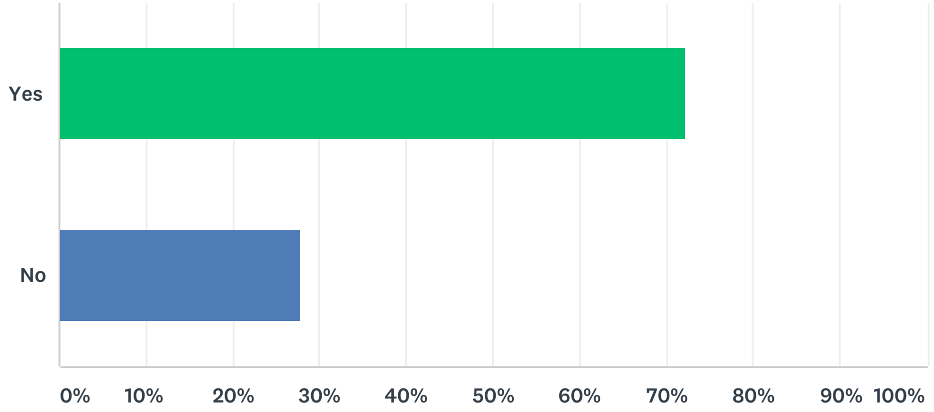

ANSWER CHOICES	RESPONSES	
Yes	72.12%	282
No	27.88%	109
TOTAL		391

Q10 Which Metabolomics Society Annual Conference(s) have you attended? Choose all that apply.

Answered: 280 Skipped: 113

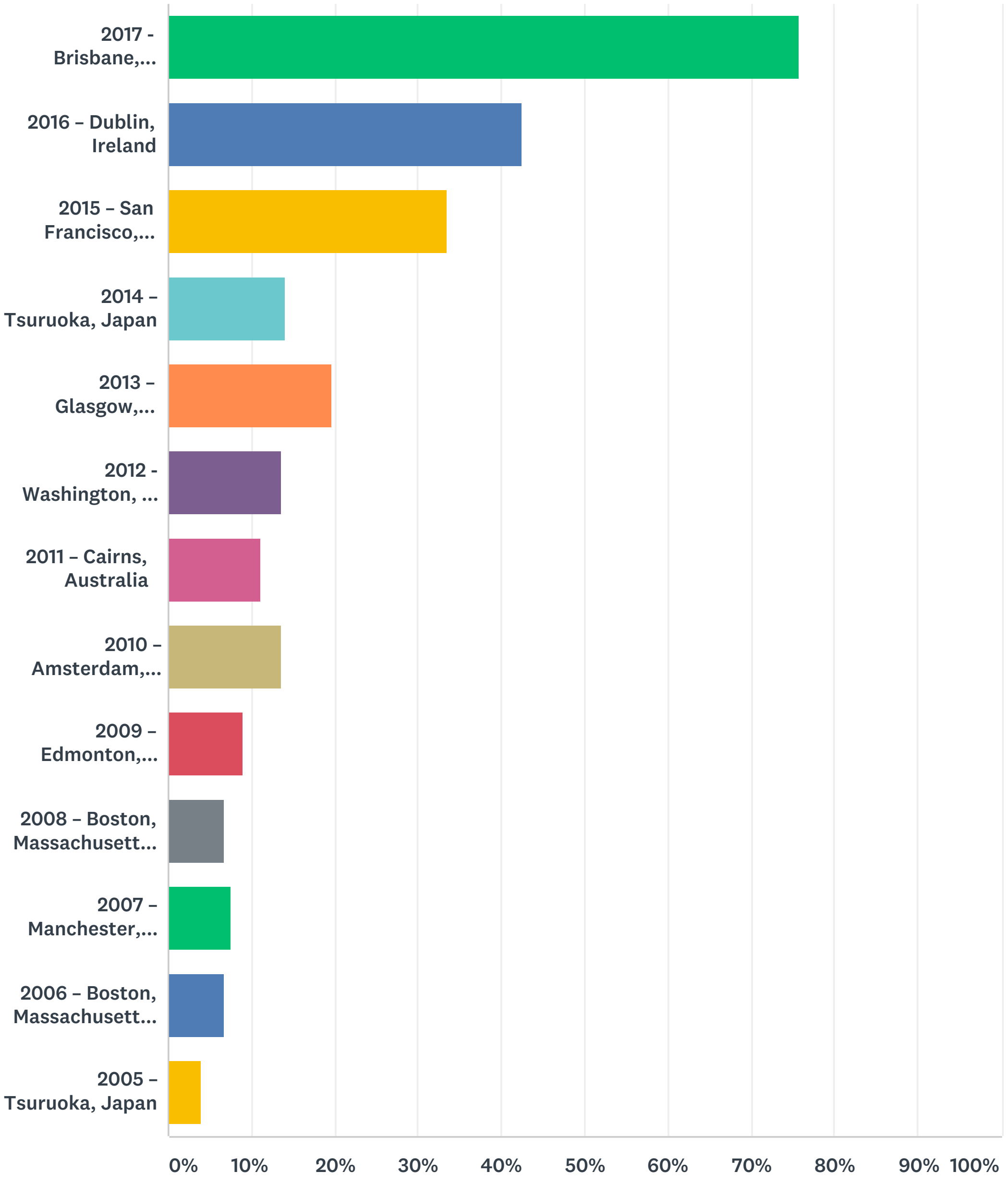

ANSWER CHOICES	RESPONSES	
2017 - Brisbane, Australia	75.71%	212
2016 – Dublin, Ireland	42.50%	119
2015 – San Francisco, California, USA	33.57%	94
2014 – Tsuruoka, Japan	13.93%	39
2013 – Glasgow, Scotland	19.64%	55

2012 - Washington, DC, USA	13.57%	38
2011 – Cairns, Australia	11.07%	31
2010 – Amsterdam, Netherlands	13.57%	38
2009 – Edmonton, Alberta, Canada	8.93%	25
2008 – Boston, Massachusetts, USA	6.79%	19
2007 – Manchester, United Kingdom	7.50%	21
2006 – Boston, Massachusetts, USA	6.79%	19
2005 – Tsuruoka, Japan	3.93%	11
Total Respondents: 280		

Q11 Do you remain a member in the years that you do not attend the annual Metabolomics Society meeting?

Answered: 278 Skipped: 115

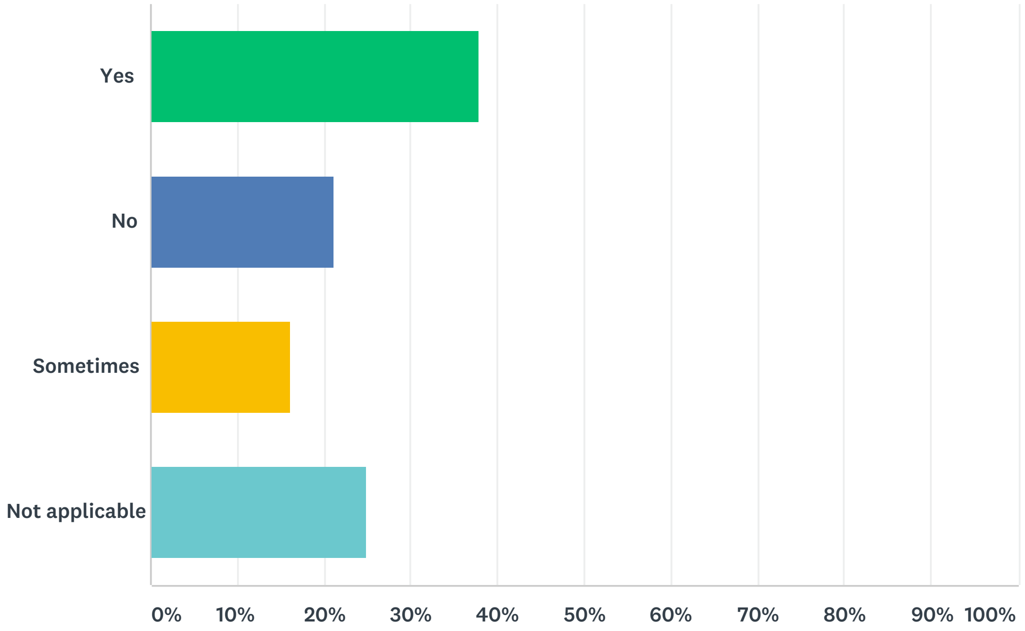

ANSWER CHOICES	RESPONSES	
Yes	37.77%	105
No	21.22%	59
Sometimes	16.19%	45
Not applicable	24.82%	69
TOTAL		278

Q12 When you attended annual Metabolomics Society meeting(s), did you also attend any of the Workshops, which generally take place the day prior to the official start of the meeting?

Answered: 152 Skipped: 241

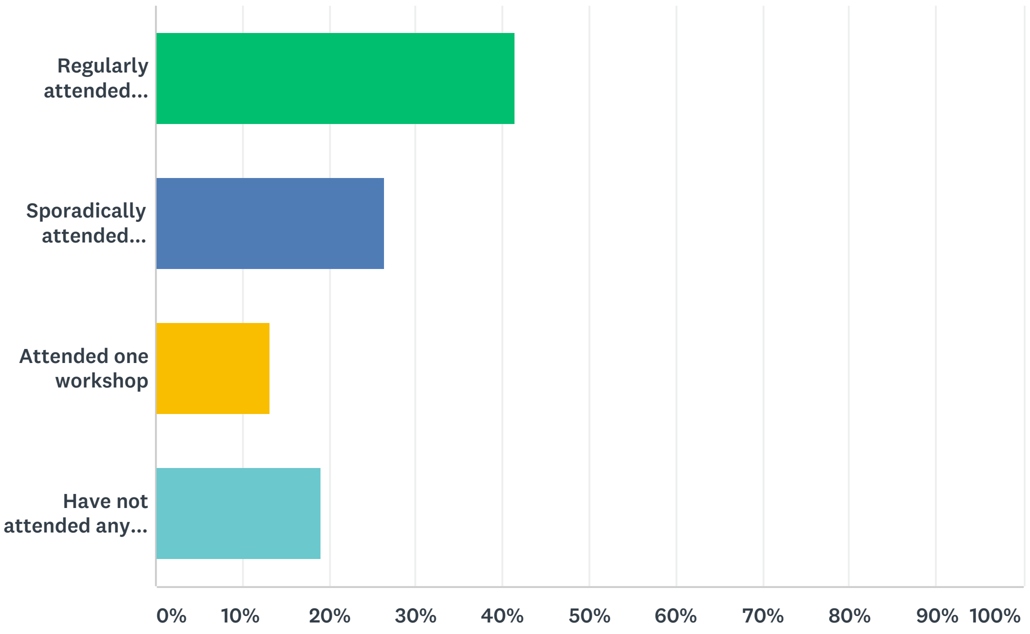

ANSWER CHOICES	RESPONSES	
Regularly attended workshops	41.45%	63
Sporadically attended workshops	26.32%	40
Attended one workshop	13.16%	20
Have not attended any workshops	19.08%	29
TOTAL		152

Q13 When you attended the Workshop(s), was it clear that the purpose of these sessions was for training?

Answered: 121   Skipped: 272

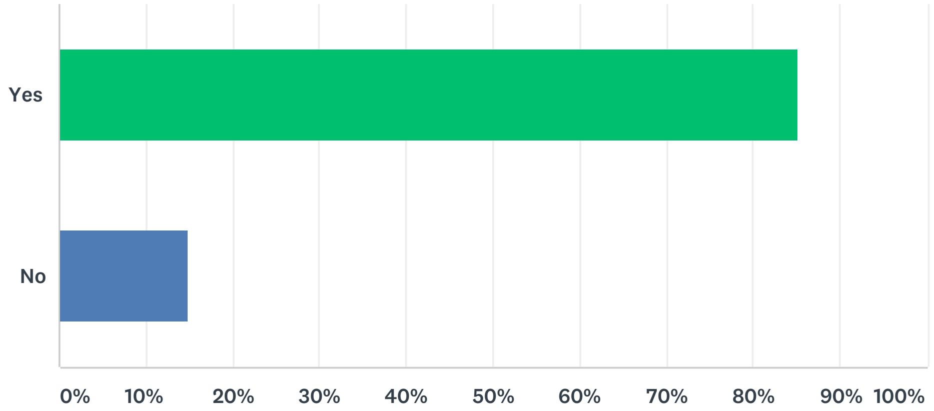

ANSWER CHOICES	RESPONSES	
Yes	85.12%	103
No	14.88%	18
TOTAL		121

Q14 Why was it not clear that the purpose of the Workshop(s) was for training?

Answered: 18 Skipped: 375

Q15 Why have you not attended the Metabolomics Society Annual Conference? Choose all that apply.

Answered: 165 Skipped: 228

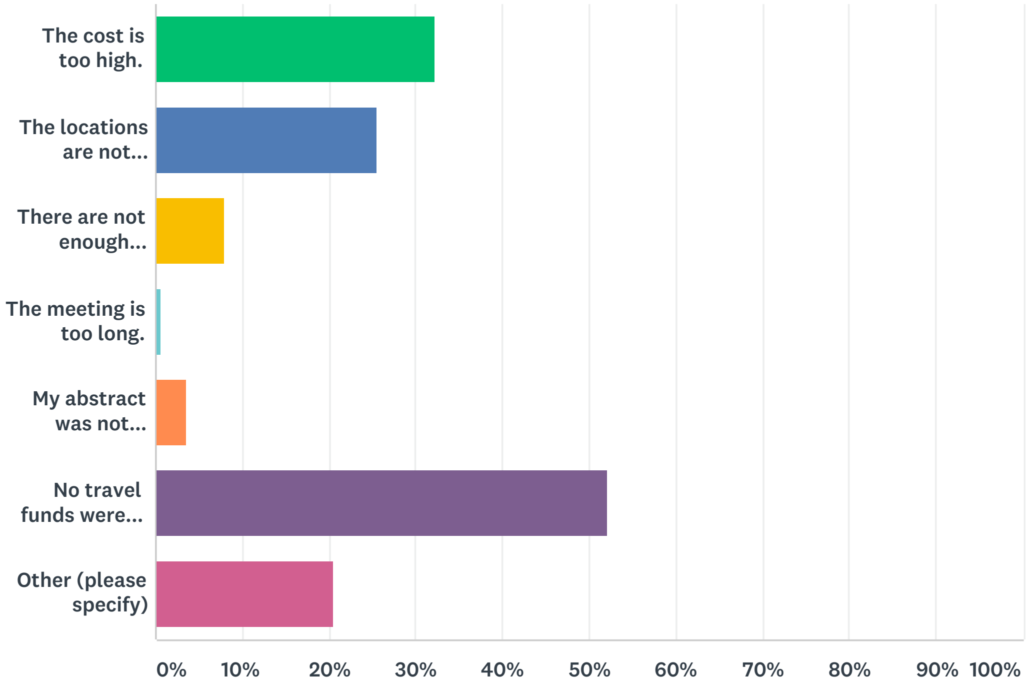

ANSWER CHOICES	RESPONSES	
The cost is too high.	32.12%	53
The locations are not convenient.	25.45%	42
There are not enough scientific sessions that interest me.	7.88%	13
The meeting is too long.	0.61%	1
My abstract was not selected for an oral presentation.	3.64%	6
No travel funds were available.	52.12%	86
Other (please specify)	20.61%	34
Total Respondents: 165		

Q16 What do you consider to be the most important benefits of being a Metabolomics Society member? Choose all that apply.

Answered: 362 Skipped: 31

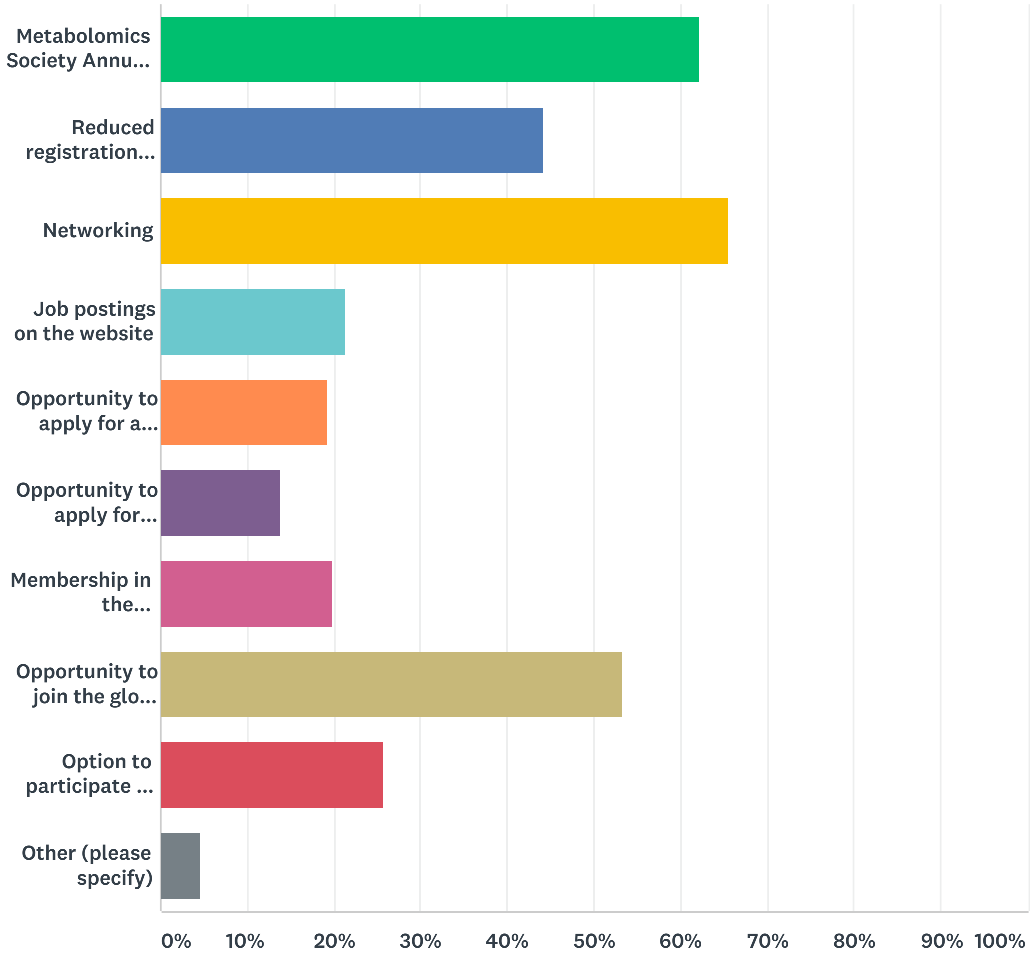

ANSWER CHOICES	RESPONSES	
Metabolomics Society Annual Meeting	62.15%	225
Reduced registration fees for the Metabolomics Society annual meeting	44.20%	160
Networking	65.47%	237
Job postings on the website	21.27%	77
Opportunity to apply for a travel award	19.34%	70
Opportunity to apply for conference support	13.81%	50
Membership in the Early-career Member Network	19.89%	72
Opportunity to join the global metabolomics community	53.31%	193
Option to participate in Metabolomics Society Task Groups (e.g. Data Quality, Data Standards, Metabolite Standard Initiative, Computational Mass Spectrometry, Model Organism Metabolomes, Precision Medicine and Pharmacometabolomics, International Affairs, Industry Engagement, and Society Strategy Task Groups)	25.69%	93

Other (please specify)	4.70%	17
Total Respondents: 362		

Q17 Have you ever participated in any of the following Metabolomics Society activities? Choose all that apply.

Answered: 206 Skipped: 187

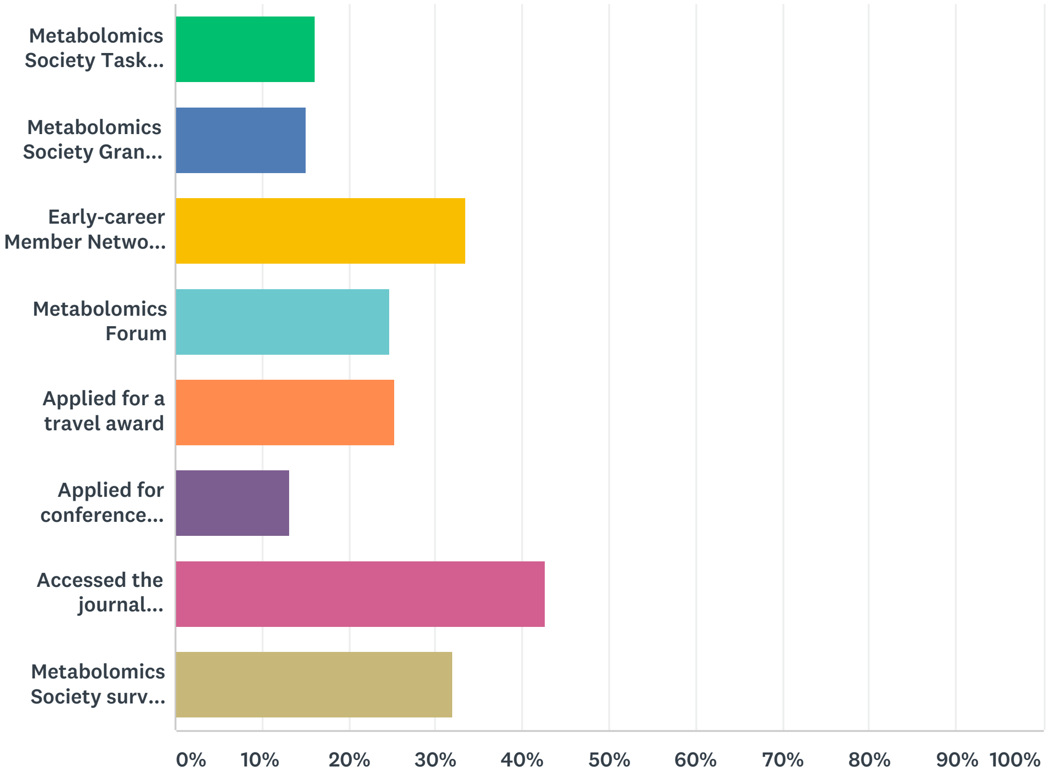

ANSWER CHOICES	RESPONSES	
Metabolomics Society Task Group	16.02%	33
Metabolomics Society Grand Challenges webinar	15.05%	31
Early-career Member Network webinar	33.50%	69
Metabolomics Forum	24.76%	51
Applied for a travel award	25.24%	52
Applied for conference support	13.11%	27
Accessed the journal "Metabolomics" through the Metabolomics Society website	42.72%	88
Metabolomics Society survey, other than the present one (e.g. Metabolomics Data Infrastructure Survey, Data Quality Task Group Survey, Training Needs in Metabolomics Survey, Computational Tools and Workflows Questionnaire)	32.04%	66
Total Respondents: 206		

Q18 What do you believe are the most important initiatives/activities that are currently supported by Metabolomics Society? Choose all that apply.

Answered: 346 Skipped: 47

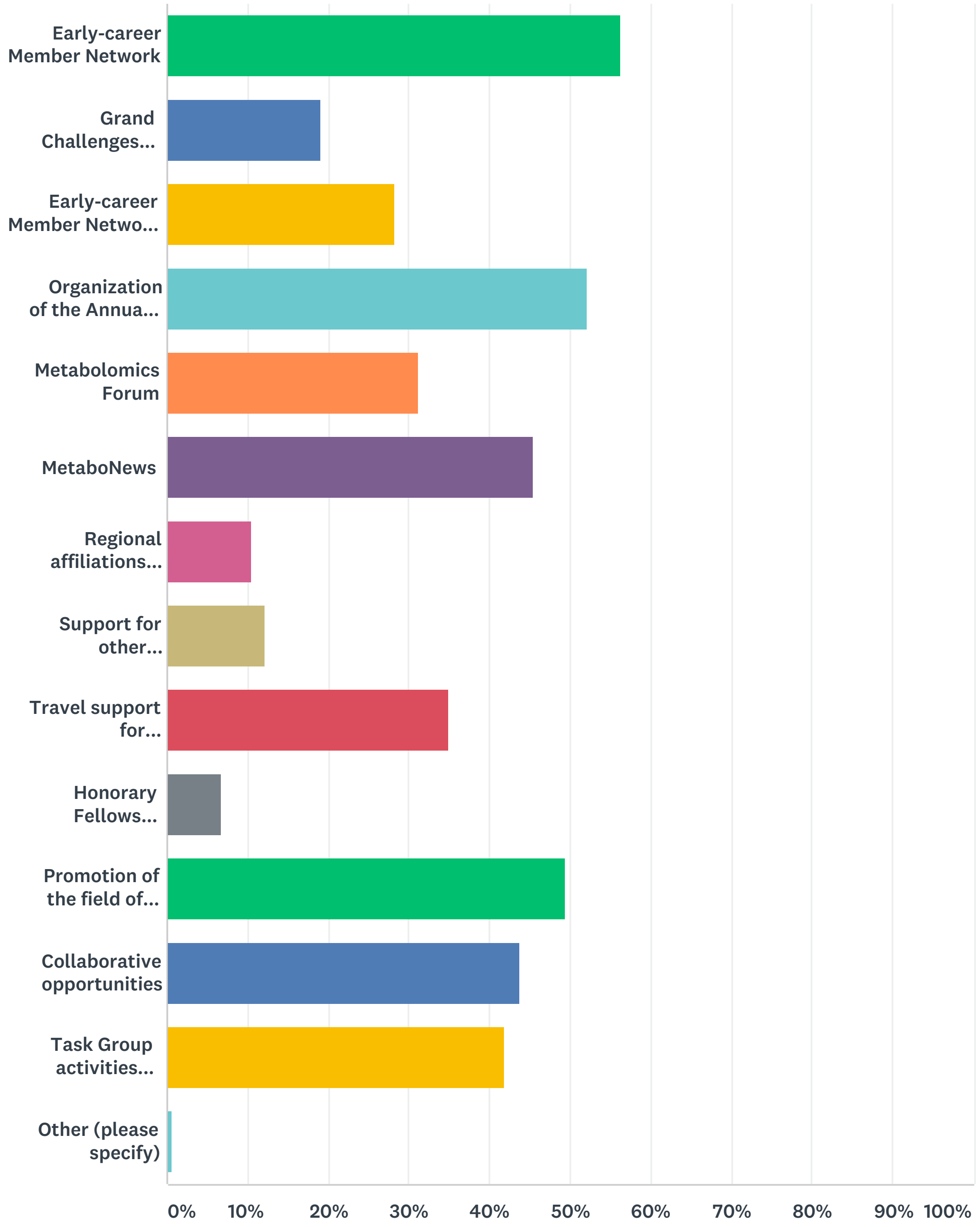

ANSWER CHOICES	RESPONSES	
Early-career Member Network	56.36%	195
Grand Challenges Webinar Series	19.08%	66
Early-career Member Network Webinar Series	28.32%	98
Organization of the Annual Meeting	52.02%	180

Metabolomics Forum	31.21%	108
MetaboNews	45.38%	157
Regional affiliations/chapters	10.40%	36
Support for other conferences	12.14%	42
Travel support for students/EMN	34.97%	121
Honorary Fellows Appointments	6.65%	23
Promotion of the field of metabolomics internationally	49.42%	171
Collaborative opportunities	43.64%	151
Task Group activities (e.g. Data Quality, Data Standards, Metabolite Standard Initiative, Computational Mass Spectrometry, Model Organism Metabolomes, Precision Medicine and Pharmacometabolomics, International Affairs, Industry Engagement, and Society Strategy Task Groups)	41.91%	145
Other (please specify)	0.58%	2
Total Respondents: 346		

Q19 What are the best strategies for the Metabolomics Society to maintain members? Choose all that apply.

Answered: 346   Skipped: 47

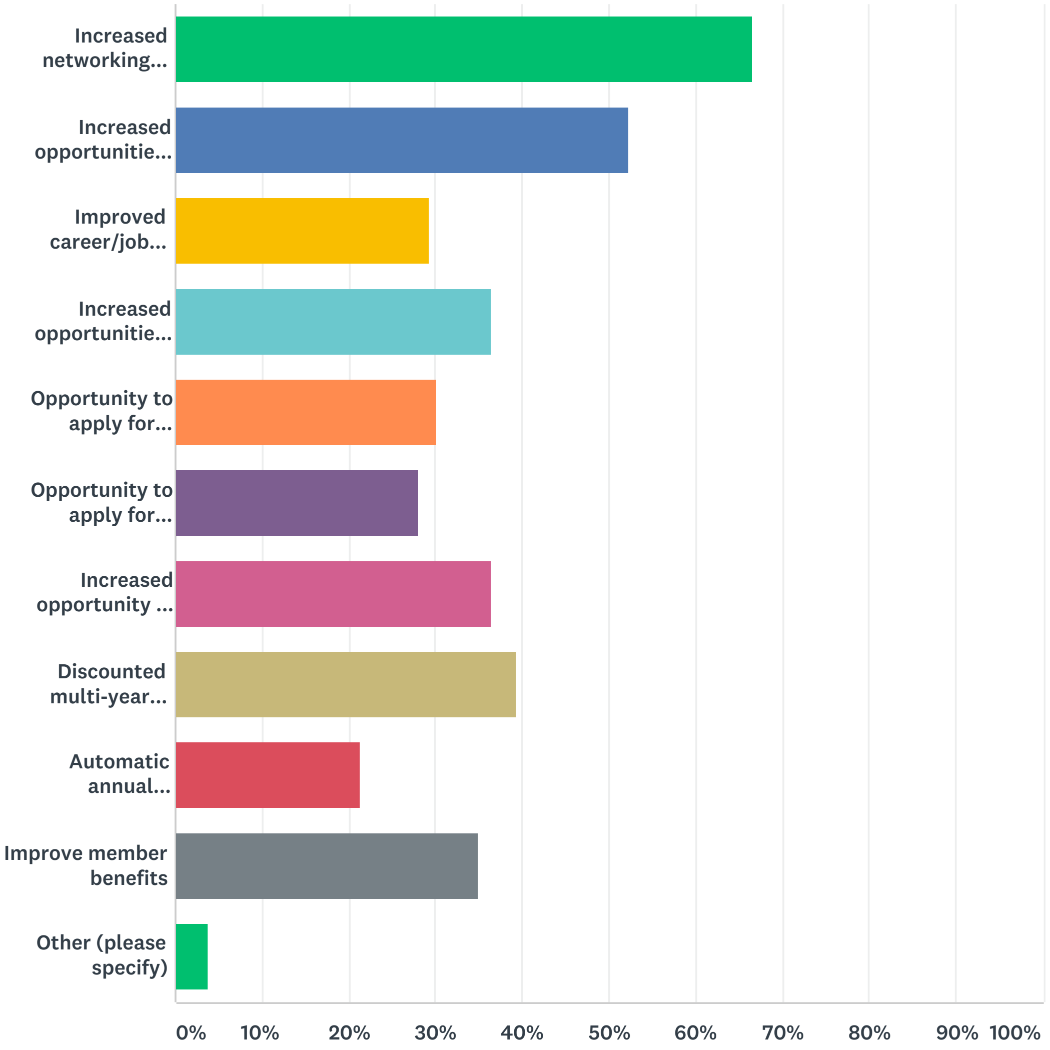

ANSWER CHOICES	RESPONSES	
Increased networking among members	66.47%	230
Increased opportunities to participate in the Annual Meetings	52.31%	181
Improved career/job postings	29.19%	101
Increased opportunities for scientific webinars	36.42%	126
Opportunity to apply for travel funds to Annual Meeting	30.06%	104
Opportunity to apply for travel funds to any metabolomics conference/meeting	28.03%	97
Increased opportunity to participate in regional/local meetings	36.42%	126
Discounted multi-year membership option	39.31%	136
Automatic annual membership renewal	21.39%	74

Improve member benefits	34.97%	121
Other (please specify)	3.76%	13
Total Respondents: 346		

Q20 What are the best strategies for the Metabolomics Society to increase membership? Choose all that apply.

Answered: 355 Skipped: 38

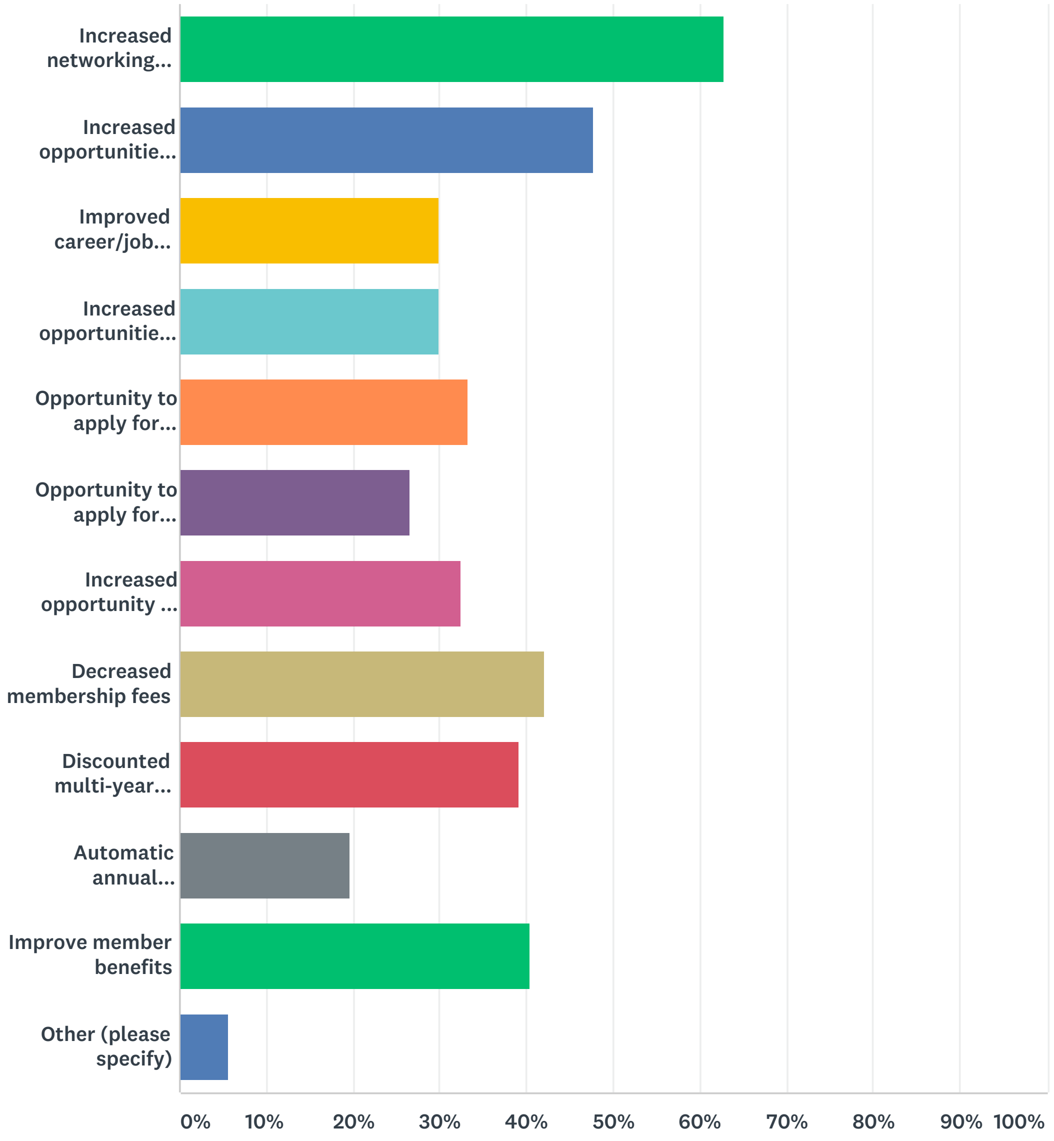

ANSWER CHOICES	RESPONSES	
Increased networking among members	62.82%	223
Increased opportunities to participate in the Annual Meetings	47.61%	169
Improved career/job postings	29.86%	106
Increased opportunities for scientific webinars	29.86%	106
Opportunity to apply for travel funds to Annual Meeting	33.24%	118
Opportunity to apply for travel funds to any metabolomics conference/meeting	26.48%	94
Increased opportunity to participate in regional/local meetings	32.39%	115

Decreased membership fees	41.97%	149
Discounted multi-year membership option	39.15%	139
Automatic annual membership renewal	19.72%	70
Improve member benefits	40.28%	143
Other (please specify)	5.63%	20
Total Respondents: 355		

Q21 What is your preferred method of communication for Metabolomics Society news and announcements?

Answered: 360 Skipped: 33

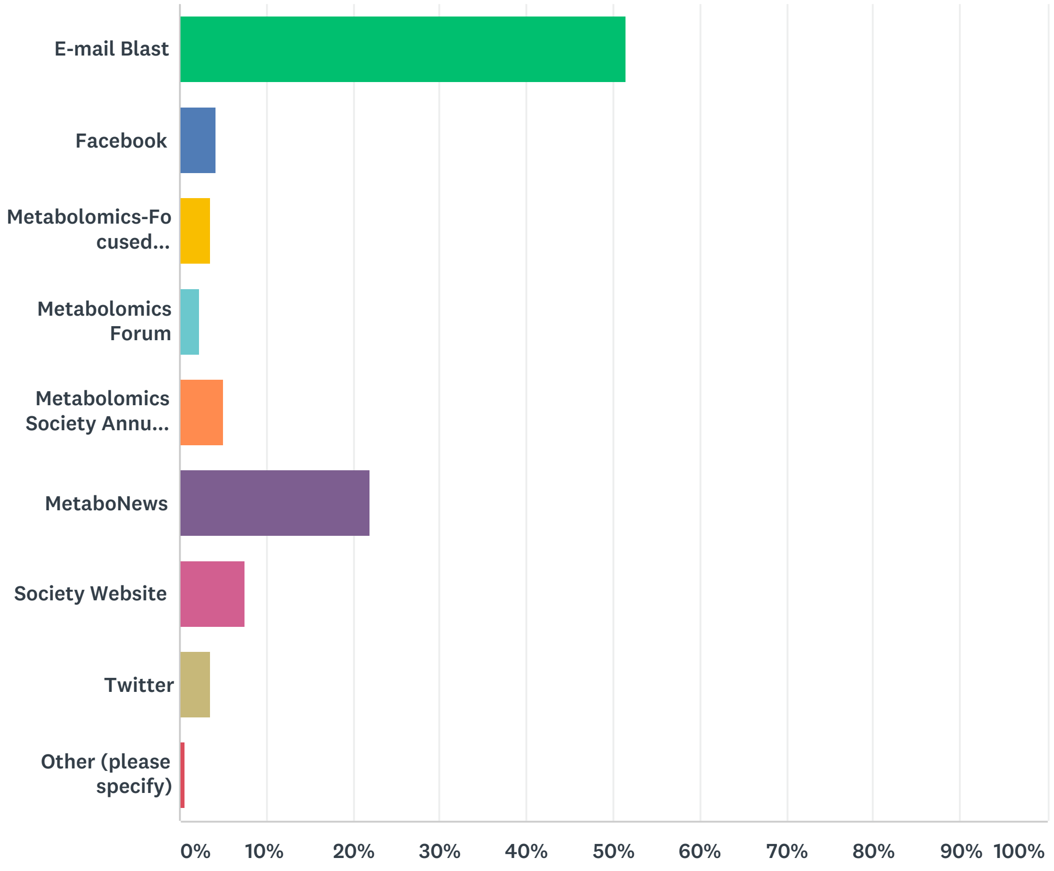

ANSWER CHOICES	RESPONSES	
E-mail Blast	51.39%	185
Facebook	4.17%	15
Metabolomics-Focused Scientific Journal	3.61%	13
Metabolomics Forum	2.22%	8
Metabolomics Society Annual Meeting	5.00%	18
MetaboNews	21.94%	79
Society Website	7.50%	27
Twitter	3.61%	13
Other (please specify)	0.56%	2
TOTAL		360

Q22 Are you a member of a Metabolomics Society affiliate? Choose all that apply.

Answered: 337 Skipped: 56

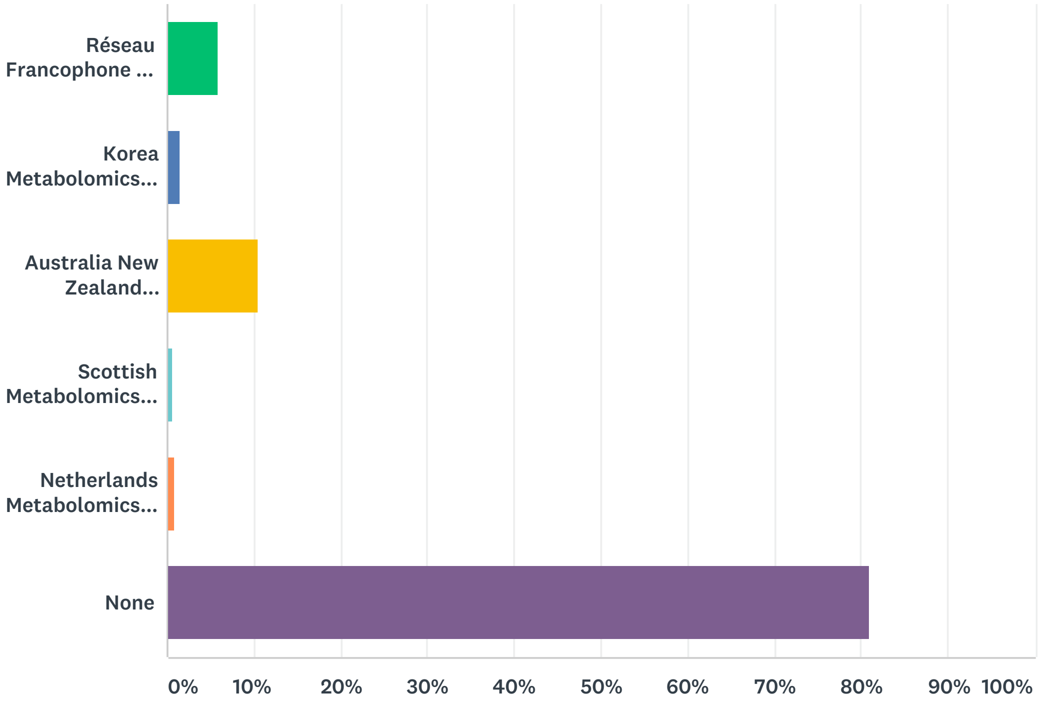

ANSWER CHOICES	RESPONSES	
Réseau Francophone de Métabolomique et Fluxomique (RFMF)	5.93%	20
Korea Metabolomics Society	1.48%	5
Australia New Zealand Metabolomics Network ((ANZMN)	10.39%	35
Scottish Metabolomics Network	0.59%	2
Netherlands Metabolomics Centre	0.89%	3
None	81.01%	273
Total Respondents: 337		

Q23 Are you a member of any other scientific societies? Choose all that apply.

Answered: 335 Skipped: 58

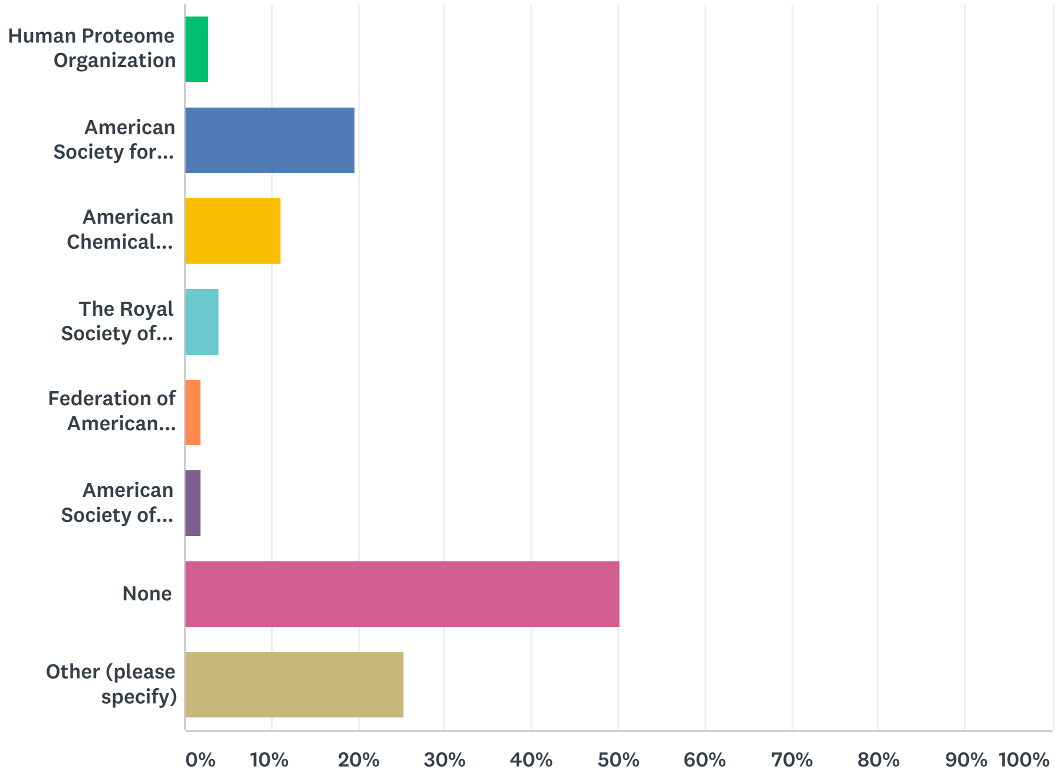

ANSWER CHOICES	RESPONSES	
Human Proteome Organization	2.69%	9
American Society for Mass Spectrometry	19.70%	66
American Chemical Society	11.04%	37
The Royal Society of Chemistry	3.88%	13
Federation of American Societies for Experimental Biology (FASEB)	1.79%	6
American Society of Plant Biology	1.79%	6
None	50.15%	168
Other (please specify)	25.37%	85
Total Respondents: 335		

Q24 What other national/international scientific meetings do you regularly attend? Choose all that apply.

Answered: 239   Skipped: 154

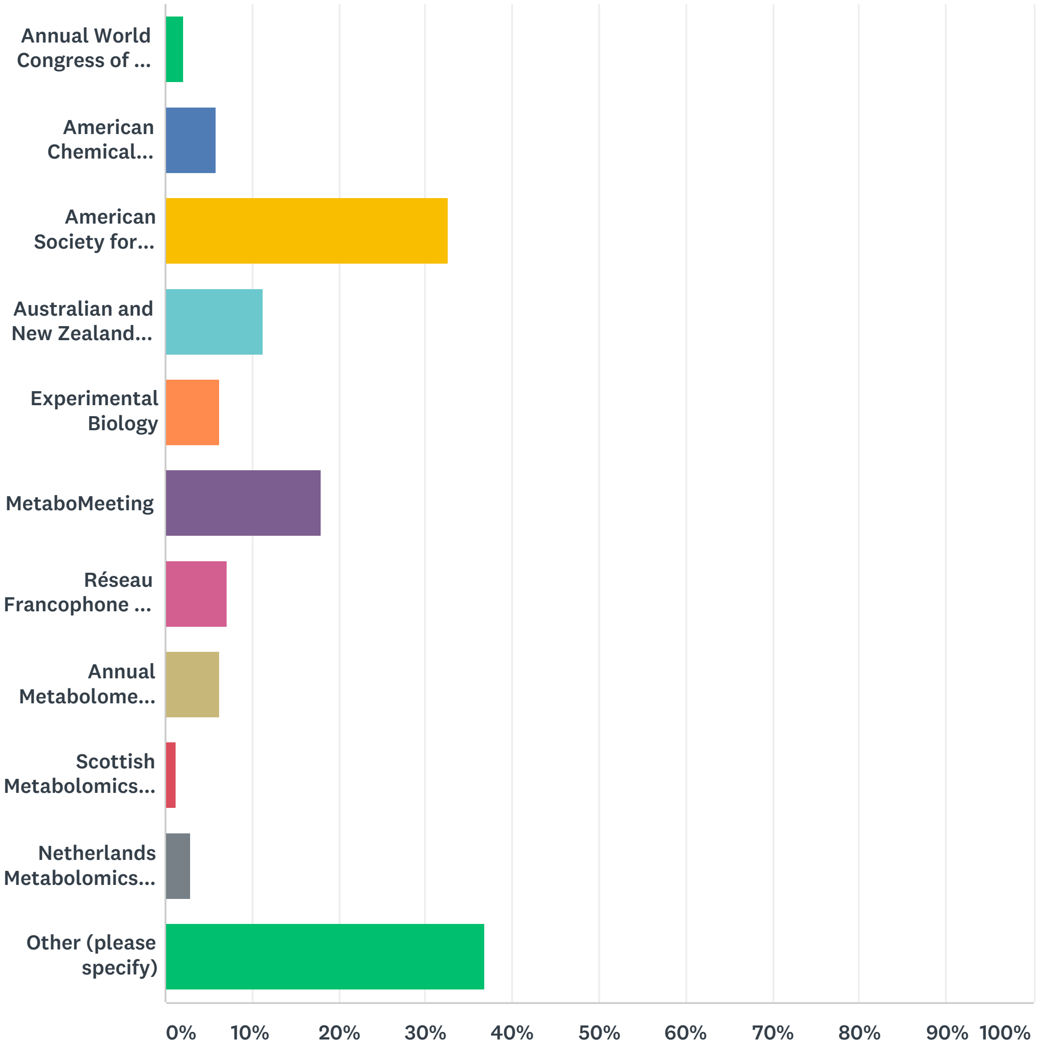

ANSWER CHOICES	RESPONSES	
Annual World Congress of the Human Proteome Organization	2.09%	5
American Chemical Society National Meeting	5.86%	14
American Society for Mass Spectrometry Annual Conference (ASMS)	32.64%	78
Australian and New Zealand Metabolomics Conference	11.30%	27
Experimental Biology	6.28%	15
MetaboMeeting	17.99%	43
Réseau Francophone de Métabolomique et Fluxomique Annual Meeting (RFMF)	7.11%	17
Annual Metabolome Symposium (Japan)	6.28%	15
Scottish Metabolomics Network	1.26%	3

Netherlands Metabolomics Society Meeting	2.93%	7
Other (please specify)	36.82%	88
Total Respondents: 239		

Q25 In which journals have you published your metabolomics research? Choose all that apply.

Answered: 327 Skipped: 66

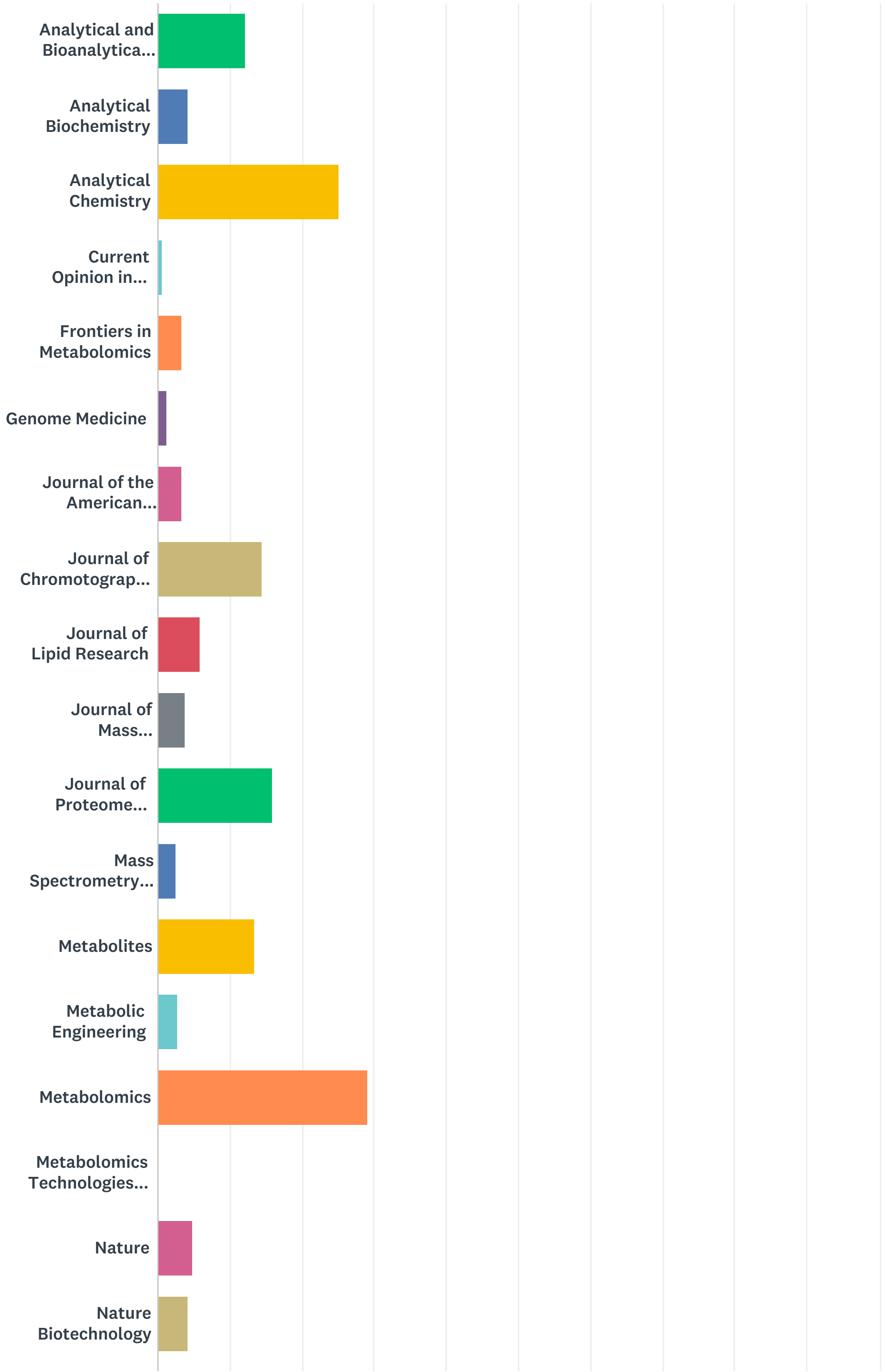

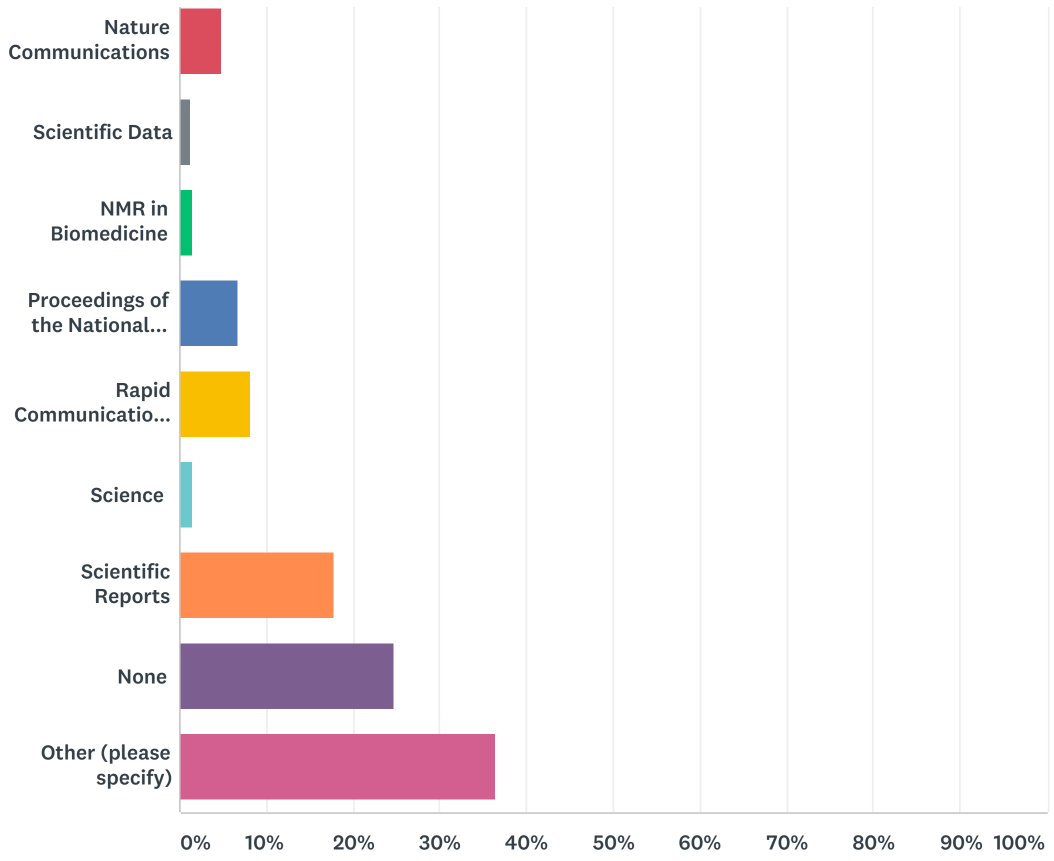

ANSWER CHOICES	RESPONSES	
Analytical and Bioanalytical Chemistry	12.23%	40
Analytical Biochemistry	4.28%	14
Analytical Chemistry	25.08%	82
Current Opinion in Lipidology	0.61%	2
Frontiers in Metabolomics	3.36%	11
Genome Medicine	1.22%	4
Journal of the American Society for Mass Spectrometry	3.36%	11
Journal of Chromotography A and B	14.37%	47
Journal of Lipid Research	5.81%	19
Journal of Mass Spectrometry	3.67%	12
Journal of Proteome Research	15.90%	52
Mass Spectrometry Reviews	2.45%	8
Metabolites	13.46%	44
Metabolic Engineering	2.75%	9
Metabolomics	29.05%	95
Metabolomics Technologies Analytical Chemistry	0.00%	0
Nature	4.89%	16

Nature Biotechnology	4.28%	14
Nature Communications	4.89%	16
Scientific Data	1.22%	4
NMR in Biomedicine	1.53%	5
Proceedings of the National Academy of Science	6.73%	22
Rapid Communications in Mass Spectrometry	8.26%	27
Science	1.53%	5
Scientific Reports	17.74%	58
None	24.77%	81
Other (please specify)	36.39%	119
Total Respondents: 327		

Q26 Is there a need for an official Metabolomics Society Journal?

Answered: 346 Skipped: 47

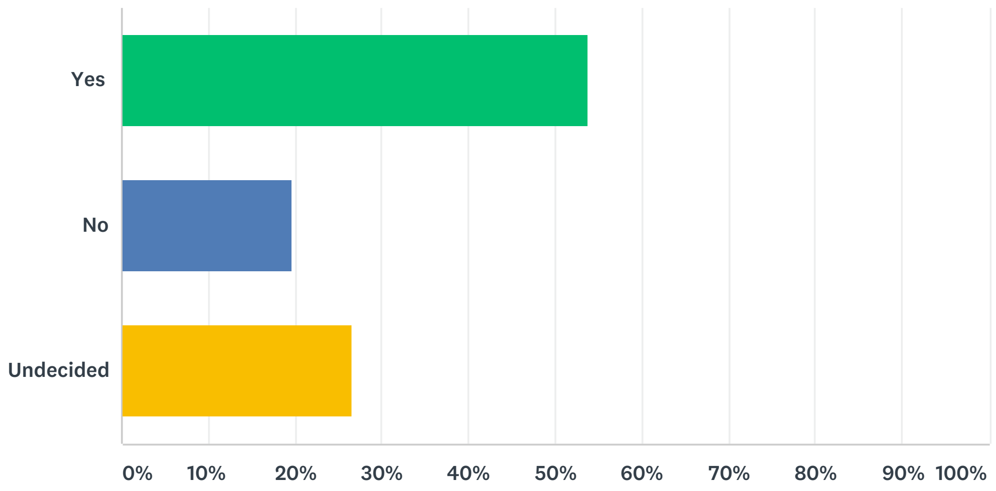

ANSWER CHOICES	RESPONSES	
Yes	53.76%	186
No	19.65%	68
Undecided	26.59%	92
TOTAL		346

Q27 Do you think the Society should partner with an existing journal?

Answered: 347   Skipped: 46

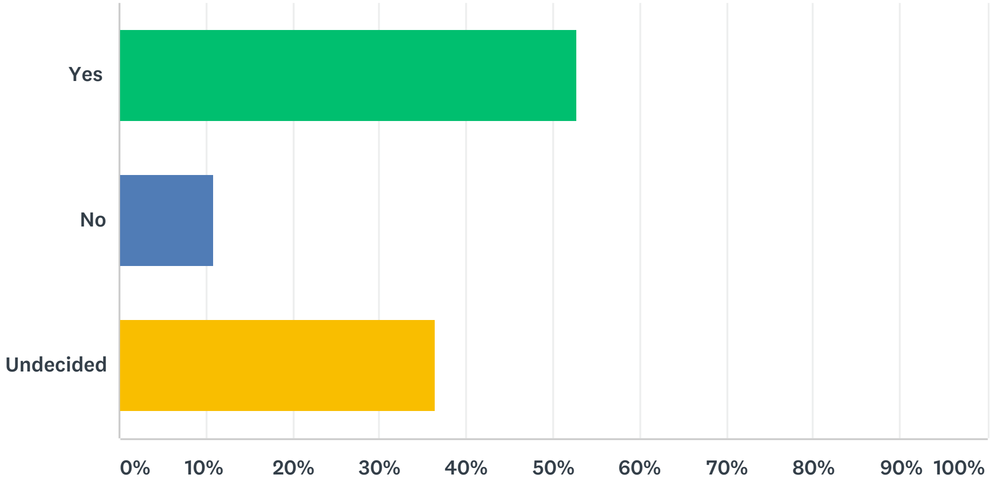

ANSWER CHOICES	RESPONSES	
Yes	52.74%	183
No	10.95%	38
Undecided	36.31%	126
TOTAL		347

Q28 If the journal publishing model was Open Access, would you choose to publish your papers for a fee (e.g. $2,500)?

Answered: 347   Skipped: 46

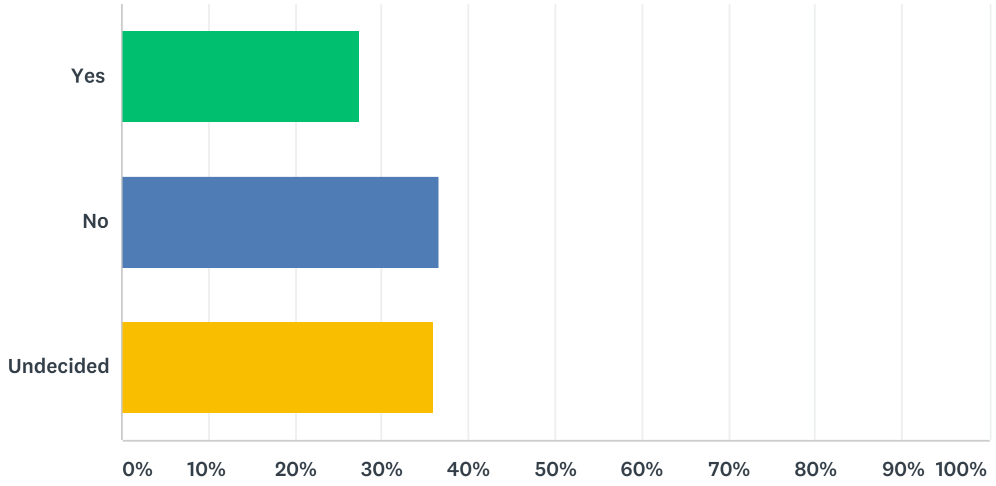

ANSWER CHOICES	RESPONSES	
Yes	27.38%	95
No	36.60%	127
Undecided	36.02%	125
TOTAL		347

Q29 Do you think that Open Access Data Archiving (MetaboLights, Metabolomics Workbench, etc) should be prerequisite to publication?

Answered: 348   Skipped: 45

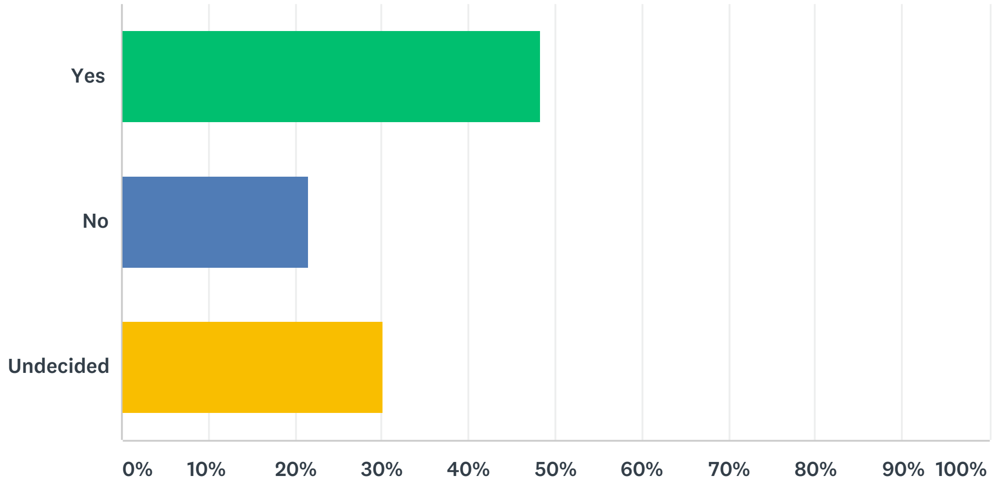

ANSWER CHOICES	RESPONSES	
Yes	48.28%	168
No	21.55%	75
Undecided	30.17%	105
TOTAL		348

Q30 What additional member benefits, if any, would you like to see the Metabolomics Society provide to maintain and/or increase membership?

Answered: 109 Skipped: 284

Q31 What types of benefits or services, training opportunities, and Metabolomics Society initiatives should be explored for implementation over the next 5-10 years?

Answered: 119 Skipped: 274

Q32 If you would like to be entered to win a $200 Amazon gift card, please provide your e-mail address.

Answered: 240 Skipped: 153
